# Temporal Trends, Seasonality, and Regional Inequalities in Perinatal Mortality in Hungary, 2000–2023: A Population-Based Ecological Study

**DOI:** 10.3390/children13101356

**Published:** 2026-10-06

**Authors:** Kristóf Németh, Tamás Lantos, Tibor András Nyári

**Affiliations:** 1 Department of Medical Physics and Informatics, Albert Szent-Györgyi Medical School, University of Szeged, 9 Korányi Alley, 6720 Szeged, Hungary; 2 Doctoral School of Experimental and Preventive Medicine, University of Szeged, 6720 Szeged, Hungary

**Keywords:** perinatal mortality, late fetal mortality, early neonatal mortality, temporal trends, seasonality, regional inequalities, maternal age, marital status, sex differences, Hungary

## Abstract

**Highlights:**

**What are the main findings?**

- Perinatal mortality declined substantially in Hungary from 2000 to 2023, with the steepest reduction observed in early neonatal mortality.
- Persistent inequalities remained despite this decline, with higher mortality among males and in Northern Hungary, alongside distinct seasonal patterns.

**What are the implications of the main findings?**

- Persistent regional and sex-related disparities indicate that improvements in perinatal outcomes have not been evenly distributed across the population.
- Continued surveillance and individual-level research integrating clinical, social, and environmental factors are needed to identify underlying mechanisms and inform targeted prevention.

**Abstract:**

Background/Objectives: Perinatal mortality reflects maternal health, quality of care, and broader social and environmental conditions. Despite long-term improvements across Europe, disparities persist. We assessed trends in perinatal, late fetal, and early neonatal mortality in Hungary during 2000–2023 and examined variation by sex, region, and maternal characteristics. Methods: This nationwide ecological study used aggregated Hungarian Central Statistical Office data on live births, late fetal deaths (≥24 gestational weeks), and early neonatal deaths (within six days). Quasi-Poisson regression models estimated incidence rate ratios (IRRs) with 95% confidence intervals. Temporal, seasonal, sex-specific, and NUTS 2 regional patterns were analyzed; maternal age and marital status were explored. Results: Among 2,235,078 live births, 10,693 late fetal and 5343 early neonatal deaths occurred. Perinatal mortality declined significantly (IRR 0.976, 95% CI 0.972–0.980), with a steeper decline in early neonatal than in late fetal mortality. Mortality during 2020–2023 exceeded counterfactual estimates based on the modeled continuation of pre-pandemic trends. Mortality was higher among males and in Northern Hungary than in Budapest. Late fetal and overall perinatal mortality peaked in May, while early neonatal mortality showed spring and autumn peaks. Mortality rates were higher among births in the youngest and oldest maternal-age groups and among those registered to mothers classified as not married. Recorded mortality attributed to intrauterine hypoxia, birth asphyxia, and fetal growth restriction increased. Conclusions: Perinatal mortality in Hungary declined substantially during 2000–2023, but sex-related and regional differences persisted. The observed seasonal, demographic, and cause-specific patterns warrant continued surveillance and further individual-level and multilevel research to investigate their underlying determinants.

## 1. Introduction

Perinatal mortality is an important and sensitive public health indicator, reflecting maternal health, the quality of antenatal, obstetric, and neonatal care, and broader socioeconomic and environmental conditions. It encompasses deaths occurring during late fetal life and the early neonatal period and provides insight into both healthcare system performance and population health inequalities.

Late fetal mortality refers to fetal deaths occurring at or beyond 24 completed weeks of gestation, when the fetus shows no signs of life after separation from the mother. This definition follows the official classification of the Hungarian Central Statistical Office (HCSO), which has used a 24-week threshold since 1997, when the boundary between early–mid and late fetal deaths was revised from 28 to 24 weeks of gestation [1,2]. For international comparison, fetal-death registration thresholds are not fully harmonized across countries and data sources; in particular, the World Health Organization (WHO) recommends a 28-week threshold for global comparability [3]. Consequently, international comparisons of stillbirth and perinatal mortality rates require caution when the underlying gestational-age definitions are not equivalent. Early neonatal mortality refers to deaths among live-born infants occurring within the first six days after birth; together, late fetal and early neonatal deaths constitute perinatal mortality [3,4]. Although these components have partly distinct etiological profiles, both are associated with maternal factors, care during pregnancy and delivery, and access to timely neonatal care [5,6,7].

Perinatal, late fetal, and neonatal mortality rates have declined substantially across most high-income European countries over recent decades, alongside advances in medical technology, changes in the organization of perinatal care, and broader implementation of preventive interventions [8]. However, recent evidence suggests that progress has slowed or plateaued in several European countries since 2015, while substantial geographic and social variation persists [9]. Globally, the neonatal period remains a major contributor to childhood mortality, with newborn deaths accounting for approximately 47% of deaths among children under five years of age and occurring predominantly in the early neonatal period and in low- and middle-income countries [4,10]. Late fetal life and the first week after birth remain particularly vulnerable periods [11]. Maternal characteristics may also contribute to variation in perinatal outcomes, with previous studies demonstrating associations between maternal age and adverse perinatal outcomes, particularly at the extremes of the maternal age distribution [12,13,14,15].

In Hungary, official statistics on perinatal, late fetal, and early neonatal mortality have been routinely collected by the HCSO for decades. Despite substantial historical improvements, recent international comparisons indicate that Hungary remains above the European average for certain perinatal mortality indicators [9]. Between 2011 and 2023, Hungary was among the countries with higher reported late fetal and perinatal mortality rates, whereas its early neonatal mortality rate was below the EU average [16]. These comparisons should be interpreted cautiously because fetal-death registration thresholds are not fully harmonized across countries.

Despite the availability of long-term national data, an integrated assessment of temporal, seasonal, sex-specific, and regional patterns in perinatal mortality in Hungary over the contemporary period is warranted. Examining these dimensions within a consistent analytical framework may provide a more detailed description of national mortality patterns and identify areas requiring further investigation.

Consequently, this study aimed to assess perinatal mortality and its main components—late fetal and early neonatal mortality—in Hungary between 2000 and 2023 using publicly available HCSO data. Specifically, we examined annual and seasonal trends, sex-specific differences, and regional variation at the NUTS 2 level. By analyzing a 24-year national time series, the study provides a population-level description of perinatal mortality patterns and a foundation for future individual-level and multilevel investigations of their underlying determinants.

## 2. Materials and Methods

### 2.1. Data Sources

This analysis was based on publicly available, aggregated birth and mortality data obtained from the Hungarian Central Statistical Office (HCSO). Mortality data are derived from civil registration records, with data collection, validation, and quality control performed by the HCSO [17]. Because this study used exclusively aggregated, anonymized secondary data from publicly accessible national databases, formal ethical approval and informed consent were not required according to national regulations and institutional guidelines.

### 2.2. Study Period and Population

The study covered all registered late fetal deaths, early neonatal deaths, and live births in Hungary between 2000 and 2023. No individual-level exclusions were applied because the analysis used aggregated national data with nationwide coverage. Perinatal mortality was defined as the sum of late fetal deaths (≥24 completed weeks of gestation) and early neonatal deaths (0–6 days after live birth), according to the Hungarian registration definition applied during the study period (see Background section for details of definitions and classification). Although more detailed gestational age categories are available in the public datasets of the Hungarian Central Statistical Office (HCSO), the present analyses were based on the aggregated gestational age grouping available in the study dataset to ensure consistency across variables and outcomes.

### 2.3. Exposures and Study Variables

Factors were classified as primary or secondary according to the focus of the analyses. Primary factors (calendar year, calendar month, sex, and NUTS 2 region) were the main variables of interest and were examined in detail, whereas secondary factors (maternal age group and marital status) were included for supplementary analyses. Maternal age was categorized into three groups: under 20 years, 20–34 years, and 35 years or older. Marital status was classified as married, never married, widowed, and divorced. For the binary comparison, never-married, widowed, and divorced mothers were combined into a broader ‘not married’ category to provide an overall comparison with married mothers. The constituent categories were also analyzed separately because the ‘not married’ group comprises socially and demographically heterogeneous populations.

Cause-of-death analyses focused on the most frequently registered perinatal causes based on the International Classification of Diseases, 10th Revision (ICD-10): fetal growth restriction and malnutrition (P05), disorders related to short gestation and low birth weight (P07), intrauterine hypoxia and birth asphyxia (P20–P21), and congenital anomalies (Q00–Q99).

### 2.4. Outcomes

The primary outcome was overall perinatal mortality. Perinatal mortality rates were calculated using total births (live births plus late fetal deaths) as the denominator. Late fetal mortality rates were likewise calculated per total births, whereas early neonatal mortality rates were calculated per live births. Secondary outcomes included late fetal mortality and early neonatal mortality, analyzed separately, as well as cause-specific perinatal mortality patterns. Mortality counts were examined using both annual and monthly aggregation.

### 2.5. Regional Classification

Regional comparisons were conducted using the second level of the European Union’s Nomenclature of Territorial Units for Statistics (NUTS 2) [18]. Hungary was divided into eight regions: Budapest (HU11), Pest County (HU12), Central Transdanubia (HU21), Western Transdanubia (HU22), Southern Transdanubia (HU23), Northern Hungary (HU31), Northern Great Plain (HU32), and Southern Great Plain (HU33). Deaths were assigned to regions according to place of birth. The Hungarian NUTS 2 regions exhibit substantial demographic and socioeconomic heterogeneity. Summary indicators of population density, maternal age, and Subnational Human Development Index (SHDI [19]) are presented in Table S1 (Supplementary Materials). Budapest is characterized by markedly higher population density, older maternal age, and higher SHDI values than the remaining regions. In contrast, Northern Hungary and the Great Plain regions generally have lower SHDI values and younger maternal age profiles.

### 2.6. Missing Data and Suppressed Cells

For data protection reasons, non-zero counts below three (i.e., one or two) are suppressed in the HCSO database. Where suppressed counts could be uniquely determined from published marginal totals, they were reconstructed algebraically. Some remaining cells that could not be uniquely determined were manually completed with values compatible with the HCSO suppression rule and the available data structure. Because the original working files did not retain a cell-level indicator distinguishing exact reconstructions from manually completed values, the numbers of cells in these two categories could not be reliably determined retrospectively.

To assess the potential influence of this uncertainty, conservative sensitivity analyses were performed for analyses based on datasets potentially affected by small-cell suppression. All completed cells with death counts of one or two were treated as potentially uncertain and were simultaneously assigned values of 1, 1.5, or 2, while cells with values of zero and counts of three or more were left unchanged. The value 1.5 was used only as a midpoint sensitivity scenario and does not represent a literal observed death count. This procedure intentionally treats all completed counts of one or two as uncertain, including values that may have been reconstructed exactly, and therefore provides a conservative assessment of the potential influence of small-cell handling. The sensitivity analyses included cause-specific annual and regional analyses and analyses by maternal age and marital status. Analyses based on datasets without suppressed death counts, including overall annual temporal trends, sex comparisons, overall regional comparisons, monthly seasonality analyses, and interrupted time-series analyses, were unaffected by small-cell suppression. The extent of unspecified information for the analyzed variables is reported separately (Supplementary Materials: Table S2). No other missing values were present in the aggregated datasets.

### 2.7. Statistical Analysis

Generalized linear models (GLMs) with a log link and quasi-Poisson variance specification were applied to examine temporal trends, seasonal effects, and regional differences. For temporal-trend analyses, calendar year was included as a continuous linear predictor; no splines or other smoothing functions were applied. The quasi-Poisson approach was used to account for overdispersion in the mortality counts.

In all models, the number of deaths served as the outcome variable. For perinatal and late fetal mortality, the logarithm of total births (live births plus late fetal deaths) was included as an offset term. In contrast, for early neonatal mortality, the logarithm of live births was used as the offset term. Results are presented as incidence rate ratios (IRRs) with 95% confidence intervals (95% CIs), with standard errors estimated under the quasi-Poisson variance specification. Statistical significance was defined as *p* < 0.05.

For the annual temporal-trend models, overdispersion was assessed using the Pearson chi-square statistic divided by the residual degrees of freedom and the residual deviance divided by the residual degrees of freedom. Residual temporal autocorrelation was evaluated using autocorrelation function (ACF) and partial autocorrelation function (PACF) plots of the deviance residuals and the Ljung–Box test at lag 4. Robustness to alternative distributional assumptions was assessed by refitting the annual temporal-trend models using negative binomial (NB) regression with the same linear time trend and population-offset specification. In addition, heteroskedasticity- and autocorrelation-consistent (HAC) Newey–West standard errors with a lag of 1 year were calculated as a sensitivity analysis to assess the robustness of statistical inference to short-range serial dependence.

The assumption of a constant linear temporal trend was additionally evaluated by comparing the primary linear quasi-Poisson model with alternative nonlinear specifications including a quadratic calendar-year term and a natural cubic spline with 3 degrees of freedom. All models used the same death-count outcome and population-offset specification as the primary temporal-trend models. Nested quasi-Poisson models were compared using F-tests, and fitted temporal trajectories were examined to assess departures from a constant linear trend. The IRRs from the primary linear models were retained as summary measures of the average annual change over the entire study period, while the nonlinear models were used to evaluate variation in the temporal trajectory.

Regional IRRs represent unadjusted geographic comparisons and were not adjusted for calendar time or for differences in sex distribution, maternal characteristics, socioeconomic conditions, demographic composition, or other population characteristics across regions.

Seasonal patterns were initially assessed using the Walter–Elwood method [20], with deaths and the corresponding population at risk aggregated by calendar month over the study period. This method accounts for differences in the population at risk across calendar months and was used to assess cyclic seasonal patterns. The Edwards geometrical test [21], which does not account for variation in population size, was additionally applied as a sensitivity analysis.

Seasonality was further evaluated using harmonic quasi-Poisson regression applied to the complete monthly time series from January 2000 through December 2023 (288 months). Monthly death counts were modeled as the outcome, with the logarithm of the corresponding monthly population at risk included as an offset (total births for late fetal and perinatal mortality and live births for early neonatal mortality). To account for the nonlinear long-term temporal trend, a natural cubic spline of continuous time with 3 degrees of freedom was included in the models. Seasonal variation was modeled using sine and cosine Fourier terms. A first harmonic with a 12-month period was used to represent an annual seasonal cycle, and a second harmonic was additionally evaluated to allow more complex within-year seasonal patterns. Nested quasi-Poisson models were compared using F-tests to assess the contribution of the first harmonic and the additional second harmonic.

Cause-specific analyses were performed for the leading causes of late fetal, early neonatal, and perinatal mortality, including fetal growth restriction and malnutrition (ICD-10 code P05), disorders of short gestation and low birth weight (P07), intrauterine hypoxia and birth asphyxia (P20–P21), and congenital anomalies (Q00–Q99). Temporal trends and regional differences in cause-specific mortality were analyzed using the same quasi-Poisson regression framework as described above, with cause-specific death counts as the outcome variable and the corresponding population at risk included as an offset term. Results are presented as incidence rate ratios (IRRs) with 95% confidence intervals and are provided in the Supplementary Materials (Supplementary Materials: Tables S3–S7). For descriptive comparison, cause-specific deaths were additionally summarized by study period as numbers, proportions of deaths within each mortality outcome, and cause-specific mortality rates per 1000 births. Total births were used as the denominator for late fetal and perinatal mortality rates, and live births for early neonatal mortality rates.

To examine whether mortality patterns changed during the COVID-19 pandemic period, interrupted time-series analyses were performed using monthly data [22], with March 2020 specified as the interruption point [23]. Seasonally adjusted quasi-Poisson regression models were fitted to monthly death counts, with the logarithm of the relevant monthly number of births included as an offset. The primary model was specified as:

log[E(Y_t_)] = log(N_t_) + β_0_ + β_1_T_t_ + β_2_I_t_ + β_3_cos(2πM_t_/12) + β_4_sin(2πM_t_/12),

where Y_t_ denotes the number of deaths in month t; N_t_ denotes the relevant population at risk (total births for late fetal and perinatal mortality and live births for early neonatal mortality); T_t_ is a continuous monthly time variable representing the underlying temporal trend; I_t_ is an indicator variable coded 0 before March 2020 and 1 from March 2020 onward; and M_t_ represents the calendar month. The coefficient β_1_ estimates the baseline temporal trend, whereas β_2_ estimates the immediate level change from March 2020. Annual seasonality was modeled using sine and cosine harmonic terms with a 12-month period.

The primary model assumed continuation of the underlying temporal slope after March 2020 and therefore did not include a post-interruption slope-change term. A supplementary model additionally included an interaction between the post-March-2020 indicator and time centered at the interruption point to assess whether allowing a change in slope improved model fit; this model was compared with the primary level-change model using an F-test.

Overdispersion was accommodated using a quasi-Poisson variance structure. Model residuals were examined over time, and residual autocorrelation was assessed using autocorrelation and partial autocorrelation function plots.

Counterfactual mortality during March 2020–December 2023 was estimated by predicting monthly deaths under a scenario in which the March 2020 level-change indicator was set to zero, while retaining the modeled baseline temporal trend, seasonal pattern, and the observed monthly population at risk. Monthly counterfactual predictions were summed over the pandemic period and compared with the corresponding observed number of deaths. The difference between observed and counterfactual deaths was expressed as both an absolute excess and a percentage relative to the counterfactual estimate. 95% confidence intervals for the cumulative counterfactual and excess deaths were derived by propagating uncertainty in the estimated regression coefficients using the variance–covariance matrix of the fitted quasi-Poisson model. The resulting estimates represent deviations from the modelled continuation of the pre-pandemic trajectory and should not be interpreted as deaths caused specifically by COVID-19.

All statistical analyses were performed using Stata version 18.0 (StataCorp LLC, College Station, TX, USA).

## 3. Results

### 3.1. Overall Patterns of Late Fetal, Early Neonatal, and Perinatal Mortality

We analyzed trends in late fetal losses, early neonatal, and perinatal mortality in Hungary over the 24 years from 2000 to 2023. During the study period, a total of 10,693 late fetal deaths and 5343 early neonatal deaths were registered.

In the same period, 2,235,078 live births occurred in Hungary, of which 1,875,985 were recorded before the COVID-19 pandemic and 359,093 during the first four years of the pandemic. Mortality counts for the main outcomes, stratified by pre-pandemic and pandemic periods, are summarized in Table 1.

Results of temporal trends, seasonal patterns, and stratified analyses are presented below.

### 3.2. Temporal Trends (Annual)

**Figure 1:**
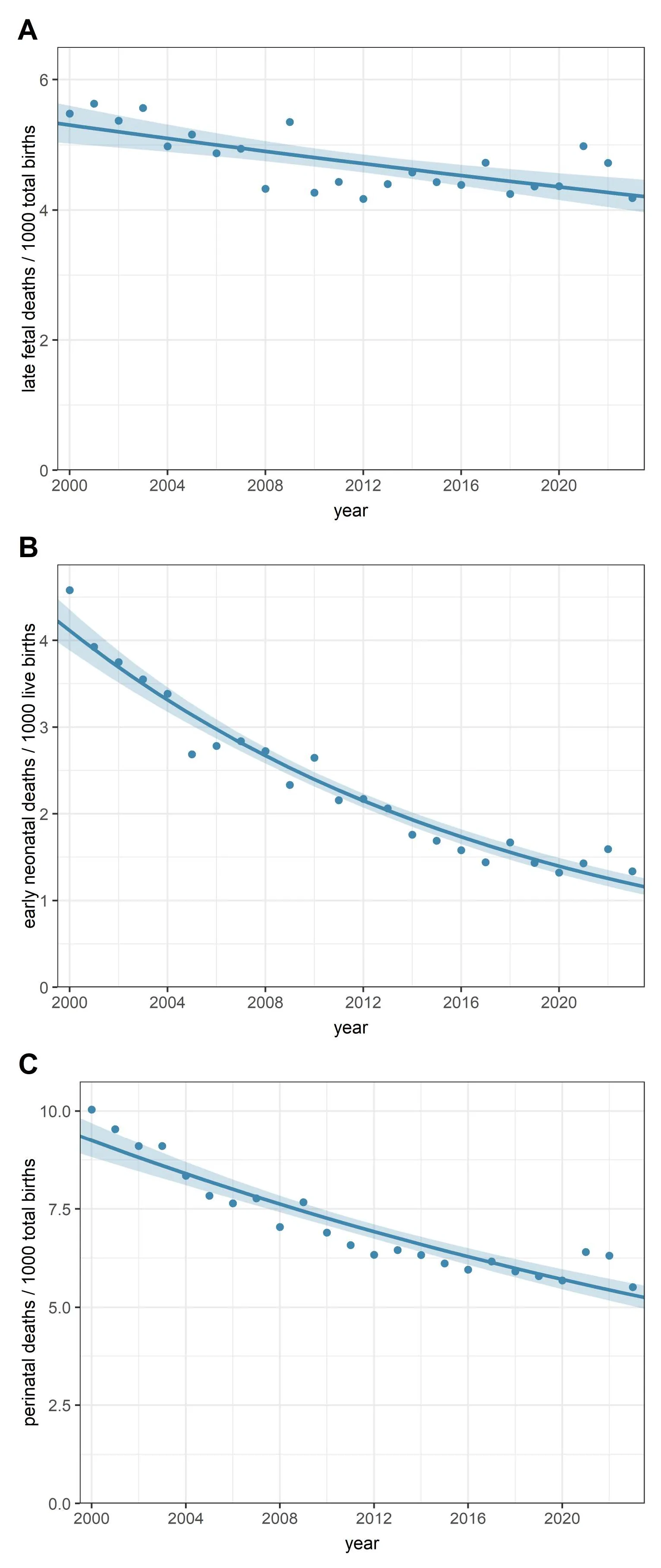
Annual trends in perinatal mortality and its components in Hungary, 2000–2023. (**A**) Late fetal mortality (≥24 gestational weeks), (**B**) early neonatal mortality (0–6 days), (**C**) overall perinatal mortality. Lines represent fitted annual trends based on quasi-Poisson regression models with total births (late fetal mortality, perinatal mortality) and live births (early neonatal mortality) as offset terms, respectively.

Quasi-Poisson regression analyses demonstrated statistically significant overall declines in all three outcomes between 2000 and 2023 (Figure 1A–C). In the primary linear models, early neonatal mortality showed the steepest average annual decline (IRR 0.947; 95% CI 0.942, 0.952), corresponding to an average annual decrease of approximately 5.3%. Late fetal mortality showed a more modest average annual decline (IRR 0.990; 95% CI 0.986, 0.994), corresponding to approximately 1.0% per year, while overall perinatal mortality declined by approximately 2.4% per year (IRR 0.976; 95% CI 0.972, 0.980). These IRRs represent average annual changes over the entire 2000–2023 period and do not imply a constant rate of decline throughout the study period.

Sensitivity analyses provided evidence of nonlinear temporal patterns for all three outcomes. Compared with the primary linear specification, inclusion of a quadratic calendar-year term significantly improved model fit for late fetal mortality (F = 10.41, *p* = 0.004), early neonatal mortality (F = 8.97, *p* = 0.007), and perinatal mortality (F = 40.60, *p* < 0.001). Natural cubic spline models with 3 degrees of freedom similarly showed significant departures from the linear specification for late fetal mortality (F = 5.35, *p* = 0.014), early neonatal mortality (F = 4.14, *p* = 0.031), and perinatal mortality (F = 19.87, *p* < 0.001). The fitted nonlinear trajectories indicated that the declines in early neonatal and overall perinatal mortality were steeper during the earlier part of the study period and became more gradual in later years. In contrast, late fetal mortality showed a more modest decline with evidence of curvature over time (Supplementary Materials: Figure S1).

The annual temporal-trend models showed evidence of overdispersion, with Pearson chi-square/df values of 2.32, 1.60, and 2.78 for late fetal, early neonatal, and perinatal mortality, respectively; the corresponding residual deviance/df values were 2.32, 1.58, and 2.74. Residual ACF/PACF plots and Ljung–Box tests showed no clear evidence of residual temporal autocorrelation for late fetal or early neonatal mortality (*p* = 0.304 and *p* = 0.883, respectively), whereas residual autocorrelation was detected for perinatal mortality (*p* = 0.015). Negative binomial sensitivity analyses yielded annual IRRs closely similar to the primary quasi-Poisson estimates. In addition, inference remained robust when Newey–West HAC standard errors were applied; the HAC-adjusted annual IRRs (95% CIs) were 0.990 (0.985, 0.995), 0.947 (0.941, 0.954), and 0.976 (0.971, 0.982) for late fetal, early neonatal, and perinatal mortality, respectively. Full model diagnostics and sensitivity analyses are presented in Table S8 and Figure S2 (Supplementary Materials).

To assess changes in perinatal mortality during the pandemic period, an interrupted time-series analysis was conducted using March 2020 as the interruption point (Supplementary Materials: Table S9; Figure S3). During March 2020–December 2023, mortality was higher than the counterfactual estimates obtained from the modeled continuation of the pre-pandemic temporal trend and seasonal pattern across all examined outcomes (Supplementary Materials: Table S9). For late fetal mortality, 1584 deaths were observed compared with 1392 counterfactual deaths, corresponding to an estimated excess of 192 deaths (95% CI 121, 264) and a relative excess of 13.8% (95% CI 8.3%, 20.0%). For early neonatal mortality, 495 deaths were observed compared with 411 counterfactual deaths, corresponding to an estimated excess of 84 deaths (95% CI 48, 119) and a relative excess of 20.4% (95% CI 10.7%, 31.6%). For overall perinatal mortality, 2079 deaths were observed compared with 1759 counterfactual deaths, corresponding to an estimated excess of 320 deaths (95% CI 244, 396) and a relative excess of 18.2% (95% CI 13.3%, 23.5%). Figure S3 (Supplementary Materials) illustrates the observed monthly mortality rates together with the model-based counterfactual trajectories. These estimates represent deviations in mortality during the pandemic period from the modelled continuation of the pre-pandemic trajectory and should not be interpreted as mortality attributable specifically to COVID-19.

### 3.3. Seasonal Patterns (Monthly)

**Figure 2:**
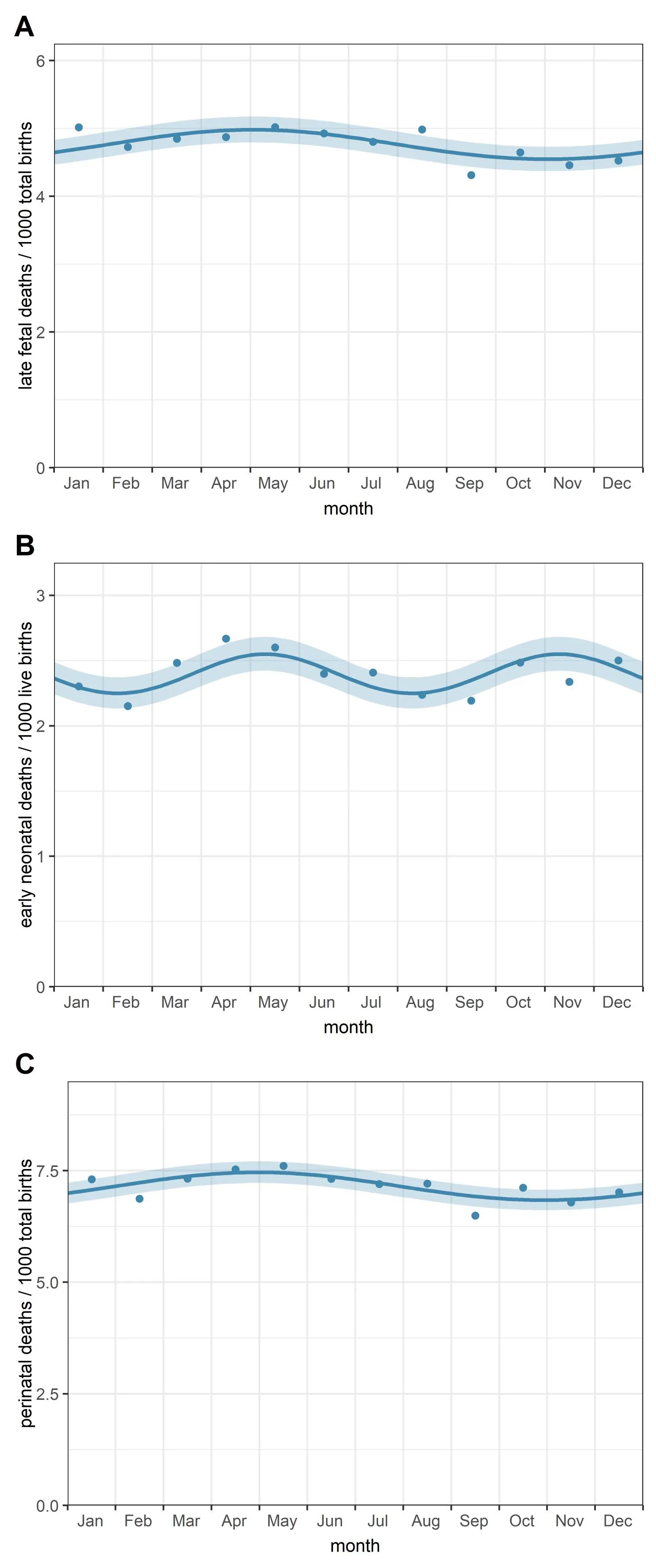
Seasonal patterns of perinatal mortality and its components in Hungary, 2000–2023. (**A**) Late fetal mortality, (**B**) early neonatal mortality, (**C**) overall perinatal mortality. Monthly distributions illustrate one- or two-peak seasonal patterns identified in regression analyses.

Seasonal variation was identified for all three mortality outcomes (Figure 2A–C).

The Walter–Elwood test indicated a unimodal seasonal pattern for late fetal mortality, with a peak in May (*p* = 0.003), and a bimodal pattern for early neonatal mortality, with peaks in May and October (*p* = 0.006). Overall perinatal mortality also showed a unimodal seasonal pattern, with the highest frequency in May (*p* < 0.001). The Edwards test (sensitivity analysis) yielded comparable evidence of seasonality.

These patterns were further evaluated using harmonic quasi-Poisson regression based on the complete monthly time series and adjusted for the nonlinear secular trend and monthly population at risk. For late fetal mortality, addition of the first 12-month harmonic significantly improved model fit compared with the secular-trend-only model (F = 4.32, *p* = 0.014), whereas addition of the second harmonic did not provide further improvement (F = 0.99, *p* = 0.372). A similar pattern was observed for perinatal mortality, for which the first harmonic improved model fit (F = 4.61, *p* = 0.011), while the additional second harmonic did not (F = 1.49, *p* = 0.228). In contrast, for early neonatal mortality, the first harmonic alone did not significantly improve model fit (F = 0.47, *p* = 0.623), whereas the addition of the second harmonic resulted in a significant improvement (F = 5.15, *p* = 0.006), supporting a more complex within-year seasonal pattern. The adjusted fitted curves were consistent with a single annual peak around May for late fetal and perinatal mortality and a bimodal pattern for early neonatal mortality, with fitted maxima around May and the October–November period.

### 3.4. Sex Differences

Across the entire study period, mortality was consistently higher among males than females for all outcomes (see Table 1). Male infants accounted for 53% of late fetal deaths, 56% of early neonatal deaths, and 54% of all perinatal deaths, with these proportions remaining stable over time.

Quasi-Poisson regression models confirmed a significantly higher mortality among males compared with females. The excess was moderate for late fetal mortality (IRR 1.06, 95% CI 1.02, 1.10), most pronounced for early neonatal mortality (IRR 1.22, 95% CI 1.16, 1.29), and remained significant for overall perinatal mortality (IRR 1.11, 95% CI 1.08, 1.15).

Sex-specific temporal trends are presented in Figure S4 (Supplementary Materials). The long-term decline in late fetal, early neonatal, and overall perinatal mortality was observed in both sexes throughout the study period. Figure S5 (Supplementary Materials) presents sex-specific regional analyses, which indicate that the overall geographic pattern of mortality was broadly similar among males and females, although mortality levels remained consistently higher among males across most regions.

### 3.5. Regional Differences by NUTS 2 Regions

**Figure 3:**
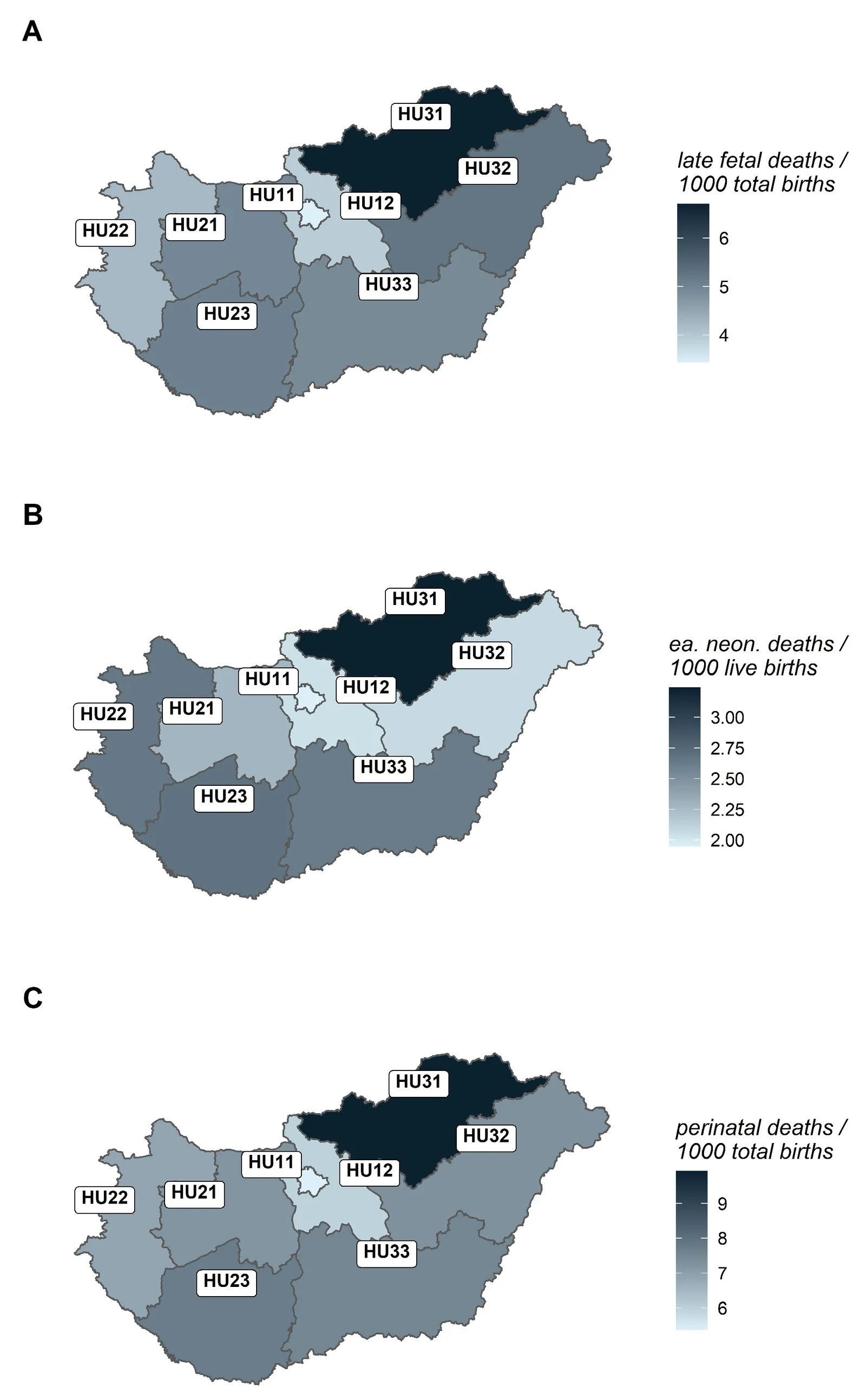
Regional distribution of perinatal mortality and its components by NUTS2 regions in Hungary, 2000–2023. (**A**) Late fetal mortality, (**B**) early neonatal mortality, (**C**) overall perinatal mortality.

Regional analyses revealed significant geographic variation in perinatal mortality and its components (Figure 3A–C). Quantitative estimates from the corresponding regression models are summarized in Table 2. Comparisons were conducted across Hungarian NUTS 2 regions, using Budapest (HU11) as the reference.

Figure 3 provides a visual overview of regional gradients, while Table 2 reports incidence rate ratios derived from quasi-Poisson regression models.

Consistently elevated mortality was observed in Northern Hungary for late fetal, early neonatal, and overall perinatal mortality. The overall pattern of regional differences was broadly similar across outcomes, with consistently elevated mortality observed in several regions compared with Budapest. However, regional disparities were generally more pronounced for late fetal mortality than for early neonatal mortality. In contrast, for early neonatal mortality, several regions—including Pest County and the Northern Great Plain—did not exhibit significantly higher mortality than Budapest.

### 3.6. Maternal Age and Marital Status (Exploratory Analyses)

Exploratory analyses assessed differences by maternal age and marital status. Compared with births registered to married mothers, mortality rates were significantly higher among births registered to mothers classified as not married across all outcomes (Supplementary Materials: Table S10). The largest difference was observed for late fetal mortality (IRR 1.91, 95% CI 1.84, 1.99), followed by perinatal mortality (IRR 1.76, 95% CI 1.71, 1.81) and early neonatal mortality (IRR 1.49, 95% CI 1.42, 1.58). However, the disaggregated analyses of never-married, widowed, and divorced categories showed heterogeneous patterns (Supplementary Materials: Table S10). Sensitivity analyses varying potentially suppressed small cell counts between 1 and 2 showed that the binary married versus not-married estimates were highly stable (Supplementary Materials: Table S11, Panel A). Estimates for the never-married and divorced categories were also stable, whereas estimates for the smaller widowed category showed greater variation across sensitivity scenarios (Supplementary Materials: Table S11, Panel B).

Significant declining trends were observed across all maternal age groups for early neonatal mortality (<20 years: IRR 0.96, 95% CI 0.95, 0.97; 20–34 years: IRR 0.95, 95% CI 0.94, 0.95; ≥35 years: IRR 0.92, 95% CI 0.91, 0.93), whereas significant declines in late fetal and perinatal mortality were observed only among mothers aged 20–34 (IRR 0.99, 95% CI 0.99, 1.00 and IRR 0.98, 95% CI 0.98, 0.98, respectively) and ≥35 years (IRR 0.96, 95% CI 0.96, 0.97 and IRR 0.95, 95% CI 0.94, 0.95, respectively); no significant trends were observed among mothers aged under 20 years (Supplementary Materials: Table S12). These annual trend estimates were unchanged to two decimal places when the potentially affected small cell counts were varied across the sensitivity scenarios.

Comparisons across broad maternal-age categories showed higher mortality rates among births in both the <20-year and ≥35-year maternal-age groups than among those in the 20–34-year reference group (Supplementary Materials: Table S13). For maternal age <20 years, IRRs were 1.55 (95% CI 1.45, 1.65) for late fetal mortality, 1.41 (95% CI 1.27, 1.55) for early neonatal mortality, and 1.50 (95% CI 1.42, 1.58) for overall perinatal mortality. For maternal age ≥35 years, the corresponding IRRs were 1.27 (95% CI 1.21, 1.33), 1.22 (95% CI 1.14, 1.31), and 1.26 (95% CI 1.21, 1.31), respectively. Analyses using the original eight maternal-age categories generally showed higher mortality rates at younger and older maternal ages relative to the 25–29-year reference group (Supplementary Materials: Table S14).

Sensitivity analyses showed that the broad maternal-age comparisons were highly stable across alternative assumptions regarding potentially suppressed small cell counts (Supplementary Materials: Table S15). Most estimates from the detailed eight-category analysis were also stable; however, estimates for the sparsest extreme-age categories, particularly 0–14 and 45–49 years, showed greater variation across sensitivity scenarios (Supplementary Materials: Table S16).

### 3.7. Trends in Major Causes of Death (Supplementary Analysis)

Despite the overall decline in perinatal mortality, trends differed across major cause-of-death categories (Supplementary Materials: Table S3). Among late fetal deaths, mortality related to fetal growth restriction and malnutrition (ICD-10 P05) increased significantly over time. Rising trends were also observed for deaths attributed to intrauterine hypoxia and birth asphyxia (P20–P21), as well as congenital anomalies.

For overall perinatal mortality, deaths associated with intrauterine hypoxia, birth asphyxia, and fetal growth restriction also showed significant increases. In contrast, no significant upward trends were detected for early neonatal deaths related to disorders of short gestation and low birth weight (P07) or congenital anomalies.

Absolute cause-specific mortality rates and proportional contributions are presented in Table S17 (Supplementary Materials). Between 2000–2019 and 2020–2023, the late fetal mortality rate attributed to P05 increased from 0.10 to 0.30 per 1000 total births, while that attributed to P20–P21 increased from 1.62 to 1.93 per 1000 total births. For early neonatal mortality, the proportional contribution of P20–P21 increased from 6.3% to 11.2%, whereas the corresponding absolute mortality rate remained 0.16 per 1000 live births. Corresponding data for categories of potentially poorly specified or residual diagnoses (e.g., “other respiratory disorders” and “unspecified conditions” within ICD-10 P00–P96), are presented in Table S18 (Supplementary Materials). Sensitivity analyses varying potentially suppressed small cell counts between 1 and 2 generally produced similar estimates for the annual cause-specific mortality trends (Supplementary Materials: Table S19). The direction of the estimated trends was unchanged across the alternative scenarios, although greater variation was observed for early neonatal mortality attributed to fetal growth restriction and malnutrition (P05).

### 3.8. Regional Distribution of Major Causes of Death (Supplementary Analysis)

Cause-specific regional analyses revealed pronounced and outcome-specific geographic differences (Supplementary Materials: Tables S4–S7). For late fetal mortality, deaths attributed to intrauterine hypoxia and birth asphyxia showed the strongest regional disparities, with markedly elevated mortality in Northern Hungary compared with Budapest. Mortality related to short gestation and low birth weight (P07) was also particularly high in Northern Hungary and Western Transdanubia.

Among early neonatal deaths, mortality due to intrauterine hypoxia and birth asphyxia was again concentrated in Northern Hungary, while deaths related to short gestation and low birth weight were most pronounced in Western Transdanubia. For overall perinatal mortality, the regional distribution of deaths due to intrauterine hypoxia and birth asphyxia closely mirrored that observed for late fetal mortality, with Northern Hungary showing consistently elevated mortality.

Sensitivity analyses under alternative assumptions for potentially suppressed small cell counts showed that most cause-specific regional estimates were substantively similar across scenarios (Supplementary Materials: Tables S20–S22). Greater variation was observed for some sparsely populated cause-specific strata, particularly in the early neonatal analyses, and these regional estimates should therefore be interpreted cautiously.

## 4. Discussion

### 4.1. Principal Findings

In this nationwide ecological study covering nearly a quarter of a century, we documented a sustained decline in perinatal, late fetal, and early neonatal mortality in Hungary. Despite these favorable long-term trends, substantial variation in mortality was observed by sex, geographic region, maternal characteristics, and cause of death.

### 4.2. Interpretation

#### 4.2.1. Temporal Trends in Context

The observed decline in perinatal mortality is consistent with patterns reported in other high-income European countries, particularly for early neonatal mortality [8]. Previous studies have attributed such favorable trends to advances in obstetric and neonatal care, including improvements in antenatal surveillance, neonatal intensive care, and the centralization of high-risk deliveries [8]. In contrast, the more modest decline observed for late fetal mortality mirrors international evidence indicating that reductions in stillbirth have progressed more slowly and remain strongly linked to maternal health, placental pathology, and broader social determinants of health [24,25]. These potential contributing factors were not directly assessed in the present study.

Recent Eurostat data provide additional European context for perinatal, late fetal, and early neonatal mortality [16]. However, the Hungarian national definition used in the present study includes fetal deaths from 24 completed weeks of gestation, whereas Eurostat international statistics may apply different criteria for late fetal deaths. Consequently, direct comparison of the absolute late fetal and perinatal mortality rates should be interpreted cautiously. Early neonatal mortality, which is defined based on deaths during the first 0–6 days after live birth, is less affected by this gestational-age comparability issue.

#### 4.2.2. Seasonality and Environmental Context

We identified statistically significant seasonal patterns across all outcomes, characterized by a consistent spring peak in late fetal and overall perinatal mortality and a bimodal pattern for early neonatal mortality. Comparable seasonal patterns have been reported in several high-income countries [26]. Previous studies have proposed several potential explanations for such seasonality, including variation in maternal infections, ambient temperature, air pollution, nutritional status, healthcare demand, and health-system strain [27,28]. These factors were not directly measured in the present study and therefore could not be evaluated as mechanisms underlying the observed seasonal variation. These patterns warrant further investigation in individual-level studies integrating clinical, environmental, and health-system data.

#### 4.2.3. Sex-Related Vulnerability in Perinatal Mortality

Male fetuses and neonates experienced consistently higher mortality across all outcomes, with the largest excess observed in early neonatal mortality. This pattern is consistent with biological explanations proposed in previous studies, including sex-specific differences in fetal growth, lung maturation, and responses to hypoxic and inflammatory stress [29,30]. Male disadvantage in perinatal outcomes has been widely documented across high-income settings, including in studies adjusting for gestational age and birth weight [31,32]. However, the mechanisms underlying the observed sex differences were not directly assessed in the present study. Our findings therefore provide descriptive evidence of persistent sex-related differences in perinatal mortality in Hungary that is consistent with the broader international literature.

#### 4.2.4. Geographic Variation

Marked regional differences in perinatal mortality were observed, with mortality rates consistently higher in Northern Hungary than in Budapest across all outcomes. These estimates represent unadjusted geographic comparisons and should not be interpreted as independent effects of region, as the regional models did not account for calendar time or differences in population composition and other potentially relevant characteristics.

Similar geographic variation in perinatal and neonatal mortality has been documented in several European countries, with higher mortality reported in socioeconomically deprived areas and regions with poorer access to specialized obstetric and neonatal services [33,34]. Previous studies have identified maternal education, income, employment status, and area-level deprivation as factors associated with perinatal outcomes and have proposed potential pathways involving delayed antenatal care, maternal comorbidities, and adverse environmental exposures [8,35,36,37]. However, these factors were not directly assessed in the present study and therefore cannot be established as explanations for the observed regional differences.

In Central and Eastern Europe, regional differences in perinatal mortality have been associated with structural disparities, including uneven distribution of healthcare resources and differential implementation of perinatal care standards [38]. The higher mortality observed in Northern Hungary is consistent with broader evidence of geographic variation in perinatal outcomes, but the factors underlying this pattern cannot be determined from the present analysis. Further studies incorporating individual- and area-level socioeconomic, clinical, demographic, and healthcare-access indicators are needed to investigate the factors underlying these regional differences.

#### 4.2.5. Maternal Characteristics and Causes of Death

Exploratory analyses showed differences in perinatal mortality by maternal age and marital status, although these findings should be interpreted cautiously given the ecological design. A U-shaped pattern was observed across the broad maternal-age categories, with higher rates of late fetal, early neonatal, and overall perinatal mortality among births in the <20-year and ≥35-year maternal-age groups than among those in the 20–34-year group. These associations were highly stable in sensitivity analyses varying potentially suppressed small cell counts between 1 and 2. Analyses using a more detailed maternal-age categorization also generally supported higher mortality at younger and older maternal ages compared with the 25–29-year reference group (Supplementary Materials: Table S14); however, sensitivity analyses indicated that estimates for the sparsest extreme-age categories, particularly 0–14 and 45–49 years, were more sensitive to assumptions regarding suppressed small cell counts and should therefore be interpreted cautiously (Supplementary Materials: Table S16). Overall, the observed age pattern is consistent with international evidence reporting higher perinatal mortality at the extremes of maternal age. Previous studies have proposed several potential explanations for these patterns, including biological, behavioral, socioeconomic, and obstetric factors [13,15,39]. These mechanisms could not be evaluated in the present aggregated analysis.

Mortality rates were also higher among births registered to mothers classified as not married than among those registered to married mothers. However, the combined ‘not married’ category comprised never-married, widowed, and divorced mothers, and the disaggregated analyses showed heterogeneous mortality patterns across these categories (Supplementary Materials: Table S10). Sensitivity analyses showed that the binary married versus not-married comparison was highly stable across alternative assumptions regarding potentially suppressed small cell counts. Estimates for the never-married and divorced categories were also stable, whereas estimates for the smaller widowed category showed greater variation and should therefore be interpreted more cautiously (Supplementary Materials: Table S11). The combined estimate should therefore be interpreted as a descriptive ecological comparison rather than as evidence of a uniform effect of unmarried status. Previous studies have reported associations between maternal marital status and socioeconomic circumstances, social support, and access to antenatal care [40,41]. These factors were not directly assessed in the present study and therefore cannot be established as explanations for the observed mortality patterns.

In addition to sociodemographic factors, cause-specific analyses indicated increasing recorded mortality rates attributed to intrauterine hypoxia, birth asphyxia, and fetal growth restriction over the study period, despite declining overall perinatal mortality. The absolute cause-specific rates and proportional contributions presented in Table S17 (Supplementary Materials) further illustrate that changes in proportional contribution do not necessarily correspond to changes of the same magnitude in population-based mortality rates. Sensitivity analyses under alternative assumptions for potentially suppressed small cell counts generally produced similar estimates for the annual cause-specific trends and most regional comparisons; however, greater variation was observed for some sparsely populated cause-specific estimates, including the annual early neonatal estimate for fetal growth restriction and malnutrition (P05) and some cause-specific regional estimates, particularly in the early neonatal analyses (Supplementary Materials: Tables S19–S22). These estimates should therefore be interpreted cautiously.

Similar changes in cause-of-death patterns have been reported in other high-income countries [42,43]. However, the present findings should be interpreted cautiously, as changes in diagnostic practices, death certification, and coding over the 24-year study period may have influenced the attribution of deaths to specific causes. Accordingly, the observed trends in recorded cause-specific mortality should not necessarily be interpreted as evidence of increasing population incidence of the underlying conditions.

#### 4.2.6. Pandemic Period (2020–2023)

Although the overall long-term downward trends in perinatal mortality remained broadly consistent, the 2020–2023 period coincided with the COVID-19 pandemic and associated changes in healthcare delivery. International studies have reported heterogeneous effects of the pandemic on stillbirth and early neonatal outcomes, potentially mediated by disruptions in antenatal care access, delayed presentation, maternal SARS-CoV-2 infection, and increased health system strain [44,45]. In this study, the pandemic period was formally evaluated using an interrupted time-series analysis comparing observed outcomes with model-based counterfactual trends. The results indicate a moderate excess in perinatal mortality during the pandemic period, with increases observed across late fetal, early neonatal, and total perinatal deaths. The interrupted time-series models (Supplementary Materials: Table S9; Figure S3) showed a divergence between observed and model-based counterfactual mortality trajectories following the onset of the pandemic, with excess mortality observed across all examined outcomes. However, these findings should be interpreted with caution. Although the analysis accounts for pre-existing temporal trends and seasonality, the use of aggregated national data limits causal inference, and the observed excess may reflect a combination of pandemic-related and other concurrent temporal changes. The pandemic period is therefore presented as a distinct contextual phase within the study timeframe rather than as a definitive causal determinant of the observed trends.

### 4.3. Strengths of the Study

This study has several important strengths. It is based on routinely collected administrative data with nationwide coverage over a 24-year period, encompassing more than 2.2 million live births and enabling the assessment of long-term temporal, seasonal, geographic, and demographic patterns in perinatal mortality in Hungary. The underlying Hungarian vital-statistics system has also demonstrated high performance in an international assessment of mortality and cause-of-death data quality [46]. The large study population, long observation period, and consistent analytic framework provide a detailed population-level description of perinatal mortality patterns and facilitate comparison across outcomes and population subgroups.

### 4.4. Limitations of the Data

This study has several limitations that should be considered when interpreting the findings. First, the ecological design based on aggregated national and regional data precludes causal inference at the individual level. Observed associations with sex, region, maternal age, and marital status may therefore reflect unmeasured confounding by correlated social, clinical, or environmental factors. In particular, the regional analyses were unadjusted geographic comparisons and did not account for calendar time or differences in sex distribution, maternal characteristics, socioeconomic conditions, demographic composition, or other population characteristics across regions. Consequently, the observed regional differences should not be interpreted as independent effects of region and may partly reflect differences in population composition or other measured and unmeasured characteristics across regions.

Second, the use of routinely collected administrative data limited the availability of detailed clinical information, such as birth weight, maternal comorbidities, smoking status, or quality-of-care indicators, which are known to influence perinatal outcomes. In addition, the available data structure did not permit simultaneous stratification by sex and calendar month; therefore, sex-specific seasonal analyses could not be performed. Information on the exact date of conception is not available in the dataset; therefore, seasonal analyses were based on the month of occurrence of the outcome, which may not fully capture potential effects related to early pregnancy exposures. Although gestational age data were available, they were not included because the analyses were based on publicly available aggregated datasets, and the primary objective was to evaluate population-level temporal, seasonal, and regional patterns. The dataset also does not allow differentiation between deaths occurring before delivery and those occurring during labor or delivery, as this information is not consistently recorded in the underlying administrative data.

Third, cause-of-death classification relies on death certification and coding practices, which may have changed over the 24-year study period and may also vary across regions. Changes in diagnostic practices, ascertainment, certification, and coding may therefore have influenced the attribution of deaths to specific causes, particularly for complex conditions such as intrauterine hypoxia, birth asphyxia, and fetal growth restriction. Moreover, changes in the proportional contribution of individual causes may partly reflect declines in competing causes rather than increases in the underlying conditions themselves. Consequently, temporal changes in recorded cause-specific mortality should not necessarily be interpreted as corresponding changes in the population incidence of the underlying conditions.

Fourth, analyses of maternal characteristics and cause-specific patterns were exploratory and based on aggregated strata; these results should therefore be interpreted as hypothesis-generating rather than confirmatory. In particular, estimates based on sparsely populated maternal-age, marital-status, or cause-specific strata may be less precise and more sensitive to the handling of small cell counts. In addition, the HCSO suppresses non-zero cell counts below three for data-protection purposes. Although suppressed counts that could be uniquely determined from published marginal totals were reconstructed algebraically, some remaining cells required manual completion. The original working files did not retain a cell-level indicator distinguishing exact reconstructions from manually completed values, precluding reliable retrospective quantification of the two categories. We therefore assessed the potential impact of this uncertainty using conservative sensitivity analyses in which all completed counts of one or two in potentially affected analyses were varied across the full range permitted by the HCSO suppression rule. The principal findings based on broad maternal-age and marital-status categories were robust to these alternative assumptions, and most cause-specific annual and regional estimates were substantively similar across sensitivity scenarios. However, greater sensitivity was observed in some sparsely populated subgroups, particularly the extreme maternal-age categories, the widowed marital-status category, and some sparse cause-specific regional comparisons. Findings from these strata should therefore be interpreted cautiously. Analyses based on datasets without suppressed death counts, including the overall temporal, sex, regional, seasonal, and interrupted time-series analyses, were not affected by this source of uncertainty.

Fifth, the mortality count data exhibited overdispersion, which was addressed using quasi-Poisson models. Diagnostic analyses showed no evidence of residual temporal autocorrelation in the annual late fetal or early neonatal mortality models, whereas residual autocorrelation was detected in the annual perinatal mortality model. Sensitivity analyses using negative binomial regression and heteroskedasticity- and autocorrelation-consistent Newey–West standard errors produced similar annual trend estimates and did not materially alter the substantive conclusions; nevertheless, residual temporal dependence may affect the precision of model-based inference. In addition, the primary annual trend models assumed a linear change in log mortality rates over calendar time. Sensitivity analyses using quadratic and natural cubic spline specifications indicated evidence of non-linearity; therefore, the linear annual IRRs should be interpreted as average changes over the full study period rather than as indicating a constant annual rate of change throughout 2000–2023.

Sixth, international comparisons of late fetal and perinatal mortality should be interpreted cautiously because gestational-age thresholds and registration criteria are not fully harmonized across countries and data sources. The Hungarian definition used in the present study includes fetal deaths from 24 completed weeks of gestation, whereas some international statistics apply a ≥28-week threshold. Inclusion of deaths occurring at 24–27 weeks may result in higher reported late fetal and perinatal mortality rates than estimates based on a ≥28-week threshold; therefore, absolute rates derived using different definitions are not directly comparable.

Finally, while the long study period allowed robust assessment of temporal and seasonal patterns, changes in clinical practice, diagnostic criteria, and registration procedures over more than two decades may have influenced observed trends.

## 5. Conclusions

Perinatal, late fetal, and early neonatal mortality declined substantially in Hungary between 2000 and 2023. Nevertheless, persistent variation was observed by sex and geographic region, with consistently higher mortality among male infants and in Northern Hungary than in Budapest. The observed seasonal patterns warrant further investigation of potential environmental and contextual determinants.

Given the ecological design and reliance on routinely collected data, these findings should be interpreted as descriptive rather than causal. The persistence of the observed differences over time highlights the importance of continued surveillance and the need for future individual-level and multilevel studies integrating clinical, social, and environmental data to better understand underlying mechanisms and inform targeted prevention strategies.

## Figures and Tables

**Table 1:** Number of late fetal deaths, early neonatal deaths, and perinatal deaths by sex and period in Hungary, 2000–2023, with corresponding numbers of live births.

Mortality Group	Sex	2000–2019, *n* (%)	2020–2023, *n* (%)	2000–2023, *n* (%)
Late fetal deaths	Male	4779 (52.8%)	878 (53.2%)	5657 (52.9%)
	Female	4265 (47.2%)	771 (46.8%)	5036 (47.1%)
	Total	9044	1649	10,693
Early neon. deaths	Male	2713 (56.1%)	301 (59.0%)	3014 (56.4%)
	Female	2120 (43.9%)	209 (41.0%)	2329 (43.6%)
	Total	4833	510	5343
Perinatal deaths	Male	7492 (54.0%)	1179 (54.6%)	8671 (54.1%)
	Female	6385 (46.0%)	980 (45.4%)	7365 (45.9%)
	Total	13,877	2159	16,036
Live births	Male	964,408 (51.4%)	184,770 (51.5%)	1,149,178 (51.4%)
	Female	911,577 (48.6%)	174,323 (48.5%)	1,085,900 (48.6%)
	Total	1,875,985	359,093	2,235,078

*Notes:* Perinatal deaths include late fetal deaths (≥24 completed weeks of gestation) and early neonatal deaths (0–6 days after live birth). Percentages are column percentages within mortality groups. Live births are shown as population denominators in the case of early neonatal deaths. Total births as population denominators in the case of late fetal deaths and perinatal deaths are calculated as the sum of live births and late fetal deaths (stillbirths).

**Table 2:** Regional differences in late fetal, early neonatal, and perinatal mortality in Hungary, 2000–2023 (reference level: Budapest, HU11).

NUTS 2 Region	Late Fetal Mortality IRR (95% CI)	Early Neonatal Mortality IRR (95% CI)	Perinatal Mortality IRR (95% CI)
Budapest (HU11)	1.00 (Reference)	1.00 (Reference)	1.00 (Reference)
Pest County (HU12)	1.14 (1.06, 1.24)	1.06 (0.95, 1.18)	1.11 (1.04, 1.18)
Central Transdanubia (HU21)	1.45 (1.34, 1.57)	1.16 (1.03, 1.29)	1.35 (1.26, 1.44)
Western Transdanubia (HU22)	1.23 (1.13, 1.34)	1.35 (1.21, 1.51)	1.28 (1.19, 1.37)
Southern Transdanubia (HU23)	1.47 (1.36, 1.60)	1.34 (1.19, 1.49)	1.43 (1.34, 1.53)
Northern Hungary (HU31)	1.95 (1.82, 2.10)	1.64 (1.48, 1.80)	1.85 (1.74, 1.96)
Northern Great Plain (HU32)	1.52 (1.42, 1.64)	1.05 (0.95, 1.16)	1.36 (1.28, 1.44)
Southern Great Plain (HU33)	1.44 (1.33, 1.55)	1.32 (1.19, 1.46)	1.40 (1.32, 1.49)

*Notes:* Incidence rate ratios (IRRs) were estimated using quasi-Poisson regression models with total births (late fetal mortality, perinatal mortality) and live births (early neonatal mortality) as offset terms, respectively.

## Data Availability

Data are available in a public, open access repository. The data used in this study are available from: https://statinfo.ksh.hu/Statinfo/themeSelector.jsp?&lang=en (accessed on 20 August 2026).

## Supplementary Materials

The following supporting information can be downloaded at https://www.mdpi.com/article/10.3390/children13101356/s1, Table S1. Regional differences in average population density, maternal age, and Subnational Human Development Index (SHDI) in Hungary, 2000–2023; Table S2. Number and proportion of missing values by each variable analyzed in late fetal, early neonatal, and perinatal mortality in Hungary, 2000–2023; Table S3. Annual trends by main death causes in late fetal, early neonatal, and perinatal mortality in Hungary, 2000–2023; Table S4. Regional differences in late fetal, early neonatal, and perinatal mortality due to fetal growth restriction and malnutrition in Hungary, 2000–2023 (reference: Budapest, HU11); Table S5. Regional differences in late fetal, early neonatal, and perinatal mortality due to disorders of short gestation and low birth weight in Hungary, 2000–2023 (reference: Budapest, HU11); Table S6. Regional differences in late fetal, early neonatal, and perinatal mortality due to intrauterine hypoxia and birth asphyxia in Hungary, 2000–2023 (reference: Budapest, HU11); Table S7. Regional differences in late fetal, early neonatal, and perinatal mortality due to congenital anomalies in Hungary, 2000–2023 (reference: Budapest, HU11); Table S8. Model diagnostics and sensitivity analyses for annual temporal trends in late fetal, early neonatal, and perinatal mortality, Hungary, 2000–2023; Table S9. Observed and model-based counterfactual mortality in late fetal deaths, early neonatal deaths, and perinatal deaths in Hungary, between March 2020 and December 2023; Table S10. Differences due to marital status in late fetal, early neonatal, and perinatal mortality in Hungary, 2000–2023; Table S11. Sensitivity analysis of mortality differences by maternal marital status under alternative assumptions for potentially suppressed small cell counts, Hungary, 2000–2023; Table S12. Annual trends in late fetal, early neonatal, and perinatal mortality in Hungary, 2000–2023; Table S13. Differences due to maternal age in late fetal, early neonatal, and perinatal mortality in Hungary, 2000–2023 (reference: 20–34 years); Table S14. Differences due to maternal age in late fetal, early neonatal, and perinatal mortality in Hungary, 2000–2023 (reference: 25–29 years); Table S15. Sensitivity analysis of mortality differences across broad maternal-age categories under alternative assumptions for potentially suppressed small cell counts, Hungary, 2000–2023; Table S16. Sensitivity analysis of mortality differences across detailed maternal-age categories under alternative assumptions for potentially suppressed small cell counts, Hungary, 2000–2023; Table S17. Absolute cause-specific mortality rates and proportional contributions of major causes to late fetal, early neonatal, and perinatal mortality by study period, Hungary, 2000–2023; Table S18. Absolute cause-specific mortality rates and proportional contributions of other respiratory disorders and unspecified causes to late fetal, early neonatal, and perinatal mortality by study period, Hungary, 2000–2023; Table S19. Sensitivity analysis of cause-specific annual mortality trends under alternative assumptions for potentially suppressed small cell counts, Hungary, 2000–2023; Table S20. Sensitivity analysis of regional differences in cause-specific late fetal mortality under alternative assumptions for potentially suppressed small cell counts, Hungary, 2000–2023; Table S21. Sensitivity analysis of regional differences in cause-specific early neonatal mortality under alternative assumptions for potentially suppressed small cell counts, Hungary, 2000–2023; Table S22. Sensitivity analysis of regional differences in cause-specific perinatal mortality under alternative assumptions for potentially suppressed small cell counts, Hungary, 2000–2023; Figure S1. Linear and nonlinear fitted temporal trends in late fetal, early neonatal, and perinatal mortality in Hungary, 2000–2023; Figure S2. Autocorrelation function (ACF) and partial autocorrelation function (PACF) plots of deviance residuals from the quasi-Poisson models of annual late fetal, early neonatal, and perinatal mortality in Hungary, 2000–2023; Figure S3. Interrupted time-series analysis of late fetal, early neonatal, and perinatal mortality in Hungary, 2000–2023; Figure S4. Annual trends in perinatal mortality and its components in Hungary, 2000–2023, stratified by sex. (A,B). Late fetal mortality (≥24 gestational weeks), (C,D). early neonatal mortality (0–6 days), (E,F). overall perinatal mortality.; Figure S5. Regional distribution of perinatal mortality and its components by NUTS 2 regions in Hungary, 2000–2023, stratified by sex. (A,B). Late fetal mortality, (C,D). early neonatal mortality, (E,F). perinatal mortality.

## Abbreviations

The following abbreviations are used in this manuscript:

ACF	Autocorrelation function
CI	Confidence interval
df	Degrees of freedom
GLM	Generalized linear model
HAC	Heteroskedasticity- and autocorrelation-consistent
HCSO	Hungarian Central Statistical Office
ICD-10	International Classification of Diseases, 10th Revision
IRR	Incidence rate ratio
NUTS	Nomenclature of Territorial Units of Statistics
PACF	Partial autocorrelation function
SHDI	Subnational Human Development Index
WHO	World Health Organization
