# Supplementary material for: Temporal Trends, Seasonality, and Regional Inequalities in Perinatal Mortality in Hungary, 2000–2023: A Population-Based Ecological Study
Source: Children (Basel). 2026 Oct 6;13(10):1356. doi: 10.3390/children13101356 (PMC13703390; doi:10.3390/children13101356)
Supplement: Supplementary file 1 [file children-13-01356-s001.zip › children-4550312-supplementary.pdf]

Table S1. Regional differences in average population density, maternal age, and Subnational Human Development Index (SHDI) in Hungary, 2000–2023.

NUTS 2 region	Pop. density (people/km^2)	Maternal age (years)	Subnational HDI (SHDI)
Budapest (HU11)	3276.6	30.9	0.916*
Pest County (HU12)	188.2	29.8	0.916*
Central Transdanubia (HU21)	98.0	29.0	0.834
Western Transdanubia (HU22)	87.2	29.4	0.853
Southern Transdanubia (HU23)	65.6	28.4	0.823
Northern Hungary (HU31)	89.3	27.2	0.805
Northern Great Plain (HU32)	84.1	27.6	0.817
Southern Great Plain (HU33)	70.7	28.9	0.829

Notes: SHDI values were available only for the former NUTS 2 region Central Hungary (former code: HU10), which comprised Budapest and Pest County. Consequently, the same SHDI values (*) were assigned to both Budapest (HU11) and Pest County (HU12). These identical values reflect data availability rather than identical levels of human development.

Table S2. Number and proportion of missing values by each variable analyzed in late fetal, early neonatal, and perinatal mortality in Hungary, 2000–2023.

Variable	Late fetal mortality: number of NAs (%)	Early neonatal mortality: number of NAs (%)	Perinatal mortality: number of NAs (%)
Calendar year	0 (0%)	0 (0%)	0 (0%)
Calendar month	0 (0%)	0 (0%)	0 (0%)
Sex	0 (0%)	0 (0%)	0 (0%)
NUTS 2 region	14 (0.13%)	10 (0.19%)	24 (0.15%)
Maternal age group	27 (0.25%)	1 (0.02%)	28 (0.17%)
Maternal marital status	29 (0.27%)	0 (0%)	29 (0.18%)

Abbreviations: NA, not available; NUTS 2, second level of the European Union's Nomenclature of Territorial Units for Statistics.

Table S3. Annual trends by main death causes in late fetal, early neonatal, and perinatal mortality in Hungary, 2000–2023.

Death cause	Late fetal mortality IRR (95% CI)	Early neonatal mortality IRR (95% CI)	Perinatal mortality IRR (95% CI)
All causes	0.99 (0.99, 0.99)	0.95 (0.94, 0.95)	0.98 (0.97, 0.98)
Fetal growth restriction and malnutrition (P05)	1.14 (1.12, 1.16)	0.87 (0.81, 0.94)	1.12 (1.10, 1.14)
Disorders of short gestation and low birth weight (P07)	0.89 (0.88, 0.91)	0.88 (0.87, 0.89)	0.88 (0.87, 0.89)
Intrauterine hypoxia and birth asphyxia (P20–P21)	1.02 (1.02, 1.03)	0.99 (0.97, 1.00)	1.02 (1.01, 1.02)
Congenital anomalies (Q00–Q99)	1.06 (1.05, 1.08)	0.97 (0.96, 0.97)	0.99 (0.98, 1.00)

Notes: Incidence rate ratios (IRRs) were estimated using quasi-Poisson regression models with total births (late fetal mortality, perinatal mortality) and live births (early neonatal mortality) as offset terms, respectively.

Table S4. Regional differences in late fetal, early neonatal, and perinatal mortality due to fetal growth restriction and malnutrition in Hungary, 2000–2023 (reference: Budapest, HU11).

NUTS 2 region	Late fetal mortality IRR (95% CI)	Early neon. mortality IRR (95% CI)	Perinatal mortality IRR (95% CI)
Budapest (HU11)	1.00 (Reference)	1.00 (Reference)	1.00 (Reference)
Pest County (HU12)	0.99 (0.62, 1.58)	0.85 (0.14, 5.10)	0.98 (0.62, 1.54)
Central Transdanubia (HU21)	1.83 (1.20, 2.80)	1.06 (0.18, 6.37)	1.78 (1.18, 2.68)
Western Transdanubia (HU22)	1.34 (0.83, 2.16)	1.23 (0.21, 7.36)	1.33 (0.84, 2.11)
Southern Transdanubia (HU23)	1.02 (0.60, 1.72)	0.65 (0.07, 6.21)	0.99 (0.59, 1.65)
Northern Hungary (HU31)	1.74 (1.15, 2.62)	1.32 (0.27, 6.53)	1.71 (1.15, 2.55)
Northern Great Plain (HU32)	0.82 (0.52, 1.31)	2.06 (0.51, 8.22)	0.91 (0.59, 1.40)
Southern Great Plain (HU33)	1.69 (1.11, 2.57)	2.30 (0.55, 9.64)	1.73 (1.16, 2.59)

Notes: Incidence rate ratios (IRRs) were estimated using quasi-Poisson regression models with live total births (late fetal mortality, perinatal mortality) and live births (early neonatal mortality) as offset terms, respectively.

Table S5. Regional differences in late fetal, early neonatal, and perinatal mortality due to disorders of short gestation and low birth weight in Hungary, 2000–2023 (reference: Budapest, HU11).

NUTS 2 region	Late fetal mortality IRR (95% CI)	Early neon. mortality IRR (95% CI)	Perinatal mortality IRR (95% CI)
Budapest (HU11)	1.00 (Reference)	1.00 (Reference)	1.00 (Reference)
Pest County (HU12)	1.16 (0.78, 1.71)	1.26 (1.01, 1.58)	1.24 (1.02, 1.50)
Central Transdanubia (HU21)	1.47 (1.00, 2.17)	1.14 (0.89, 1.45)	1.22 (0.99, 1.50)
Western Transdanubia (HU22)	1.29 (0.85, 1.96)	1.74 (1.39, 2.18)	1.62 (1.33, 1.98)
Southern Transdanubia (HU23)	0.99 (0.62, 1.57)	1.62 (1.28, 2.04)	1.45 (1.18, 1.79)
Northern Hungary (HU31)	3.32 (2.42, 4.57)	1.07 (0.84, 1.35)	1.63 (1.36, 1.96)
Northern Great Plain (HU32)	0.56 (0.36, 0.88)	0.86 (0.68, 1.08)	0.78 (0.64, 0.96)
Southern Great Plain (HU33)	2.19 (1.55, 3.09)	1.38 (1.11, 1.72)	1.58 (1.32, 1.91)

Notes: Incidence rate ratios (IRRs) were estimated using quasi-Poisson regression models with live total births (late fetal mortality, perinatal mortality) and live births (early neonatal mortality) as offset terms, respectively.

Table S6. Regional differences in late fetal, early neonatal, and perinatal mortality due to intrauterine hypoxia and birth asphyxia in Hungary, 2000–2023 (reference: Budapest, HU11).

NUTS 2 region	Late fetal mortality IRR (95% CI)	Early neon. mortality IRR (95% CI)	Perinatal mortality IRR (95% CI)
Budapest (HU11)	1.00 (Reference)	1.00 (Reference)	1.00 (Reference)
Pest County (HU12)	1.60 (1.33, 1.92)	1.11 (0.73, 1.70)	1.51 (1.27, 1.79)
Central Transdanubia (HU21)	3.50 (2.96, 4.13)	1.01 (0.63, 1.60)	3.03 (2.60, 3.54)
Western Transdanubia (HU22)	2.07 (1.71, 2.51)	1.20 (0.76, 1.91)	1.91 (1.60, 2.28)
Southern Transdanubia (HU23)	2.22 (1.84, 2.69)	1.22 (0.77, 1.94)	2.04 (1.71, 2.42)
Northern Hungary (HU31)	7.58 (6.52, 8.81)	2.15 (1.49, 3.10)	6.56 (5.72, 7.53)
Northern Great Plain (HU32)	4.56 (3.91, 5.32)	1.43 (0.98, 2.09)	3.98 (3.46, 4.57)
Southern Great Plain (HU33)	2.40 (2.02, 2.86)	1.26 (0.83, 1.92)	2.19 (1.87, 2.57)

Notes: Incidence rate ratios (IRRs) were estimated using quasi-Poisson regression models with live total births (late fetal mortality, perinatal mortality) and live births (early neonatal mortality) as offset terms, respectively.

Table S7. Regional differences in late fetal, early neonatal, and perinatal mortality due to congenital anomalies in Hungary, 2000–2023 (reference: Budapest, HU11).

NUTS 2 region	Late fetal mortality IRR (95% CI)	Early neon. mortality IRR (95% CI)	Perinatal mortality IRR (95% CI)
Budapest (HU11)	1.00 (Reference)	1.00 (Reference)	1.00 (Reference)
Pest County (HU12)	1.07 (0.71, 1.62)	0.89 (0.70, 1.13)	0.93 (0.76, 1.15)
Central Transdanubia (HU21)	1.34 (0.89, 2.02)	1.06 (0.82, 1.35)	1.12 (0.91, 1.39)
Western Transdanubia (HU22)	1.14 (0.73, 1.79)	1.00 (0.77, 1.30)	1.03 (0.82, 1.30)
Southern Transdanubia (HU23)	1.01 (0.63, 1.62)	1.39 (1.09, 1.78)	1.30 (1.05, 1.61)
Northern Hungary (HU31)	1.08 (0.71, 1.63)	1.53 (1.23, 1.89)	1.42 (1.17, 1.71)
Northern Great Plain (HU32)	0.64 (0.41, 1.00)	1.00 (0.80, 1.24)	0.91 (0.74, 1.11)
Southern Great Plain (HU33)	1.60 (1.10, 2.34)	1.25 (1.00, 1.57)	1.33 (1.10, 1.62)

Notes: Incidence rate ratios (IRRs) were estimated using quasi-Poisson regression models with live total births (late fetal mortality, perinatal mortality) and live births (early neonatal mortality) as offset terms, respectively.

Table S8. Model diagnostics and sensitivity analyses for annual temporal trends in late fetal, early neonatal, and perinatal mortality, Hungary, 2000–2023.

Statistical measure	Late fetal mortality	Early neonatal mortality	Perinatal mortality
Pearson χ^2/df	2.32	1.60	2.78
Deviance/df	2.32	1.58	2.74
Ljung–Box p-value	0.304	0.883	0.015
Quasi-Poisson IRR (95% CI)	0.9901 (0.9860, 0.9943)	0.9473 (0.9425, 0.9522)	0.9761 (0.9724, 0.9797)
Negative binomial IRR (95% CI)	0.9903 (0.9864, 0.9943)	0.9479 (0.9434, 0.9525)	0.9768 (0.9733, 0.9803)
HAC-adjusted IRR (95% CI)	0.9901 (0.9855, 0.9948)	0.9473 (0.9409, 0.9538)	0.9761 (0.9705, 0.9816)

Notes: The primary annual temporal-trend analyses were fitted using quasi-Poisson regression with death counts as the outcome and the logarithm of the relevant population at risk as an offset. Pearson χ^2/df and residual deviance/df were used to assess overdispersion. Residual temporal autocorrelation was evaluated using ACF/PACF plots of deviance residuals and the Ljung–Box test at lag 4. As sensitivity analyses, the annual trends were additionally estimated using negative-binomial regression with the same linear time trend and population-offset specification and using heteroskedasticity- and autocorrelation-consistent (HAC) Newey–West standard errors with a lag of 1 year. IRRs represent the multiplicative change in the mortality rate associated with a one-year increase in calendar year. CI, confidence interval; HAC, heteroskedasticity- and autocorrelation-consistent; IRR, incidence rate ratio.

Table S9. Observed and model-based counterfactual mortality in late fetal deaths, early neonatal deaths, and perinatal deaths in Hungary, between March 2020 and December 2023.

Mortality group	Observed deaths, n	Counterfactual deaths, n (95% CI)	Excess deaths, n (95% CI)	Excess, % (95% CI)
Late fetal deaths	1584	1392 (1320, 1463)	192 (121, 264)	13.8 (8.3%, 20.0%)
Early neon. deaths	495	411 (376, 447)	84 (48, 119)	20.4% (10.7%, 31.6%)
Perinatal deaths	2079	1759 (1683, 1835)	320 (244, 396)	18.2% (13.3%, 23.5%)

Notes: Counterfactual deaths represent the estimated number of deaths expected during March 2020–December 2023 under continuation of the pre-pandemic temporal trend and seasonal pattern, based on seasonally adjusted quasi-Poisson interrupted time-series models with March 2020 as the interruption point. Excess deaths were calculated as observed minus counterfactual deaths. Percentage excess was calculated relative to the counterfactual estimate. 95% confidence intervals reflect uncertainty in the model-based counterfactual estimates. These estimates represent deviations from the modelled pre-pandemic trajectory and should not be interpreted as deaths attributable specifically to COVID-19.

Table S10. Differences due to marital status in late fetal, early neonatal, and perinatal mortality in Hungary, 2000–2023.

Marital status	Late fetal mortality IRR (95% CI)	Early neon. mortality IRR (95% CI)	Perinatal mortality IRR (95% CI)
Married	1.00 (Reference)	1.00 (Reference)	1.00 (Reference)
Not married	1.91 (1.84, 1.99)	1.49 (1.42, 1.58)	1.76 (1.71, 1.81)
Never married	2.05 (1.97, 2.13)	1.44 (1.36, 1.52)	1.82 (1.77, 1.88)
Widowed	1.49 (1.04, 2.11)	2.53 (1.77, 3.63)	1.86 (1.45, 2.40)
Divorced	0.82 (0.72, 0.93)	1.89 (1.69, 2.11)	1.21 (1.11, 1.31)

Notes: Incidence rate ratios (IRRs) were estimated using quasi-Poisson regression models with live total births (late fetal mortality, perinatal mortality) and live births (early neonatal mortality) as offset terms, respectively.

Table S11. Sensitivity analysis of mortality differences by maternal marital status under alternative assumptions for potentially suppressed small cell counts, Hungary, 2000–2023.

A. Binary comparison of maternal marital status: not married versus married.

**Mortality group**	**Current IRR (95% CI)**	**All 1 IRR (95% CI)**	**All 1.5 IRR (95% CI)**	**All 2 IRR (95% CI)**
Late fetal deaths	1.91 (1.84, 1.99)	1.91 (1.84, 1.98)	1.91 (1.84, 1.99)	1.91 (1.84, 1.99)
Early neon. deaths	1.49 (1.42, 1.58)	1.49 (1.42, 1.58)	1.50 (1.42, 1.58)	1.50 (1.42, 1.58)
Perinatal deaths	1.76 (1.71, 1.81)	1.76 (1.70, 1.81)	1.76 (1.71, 1.81)	1.76 (1.71, 1.82)

B. Disaggregated comparison of maternal marital status: never-married, widowed, and divorced versus married.

**Mortality group**	**Marital status**	**Current IRR (95% CI)**	**All 1 IRR (95% CI)**	**All 1.5 IRR (95% CI)**	**All 2 IRR (95% CI)**
Late fetal deaths	Never-married	2.05 (1.97, 2.13)	2.05 (1.97, 2.13)	2.05 (1.97, 2.13)	2.05 (1.97, 2.13)
	Widowed	1.49 (1.04, 2.11)	1.29 (0.89, 1.89)	1.51 (1.06, 2.14)	1.73 (1.24, 2.39)
	Divorced	0.82 (0.72, 0.93)	0.81 (0.71, 0.92)	0.81 (0.72, 0.92)	0.82 (0.72, 0.93)

Mortality group	Marital status	Current IRR (95% CI)	All 1 IRR (95% CI)	All 1.5 IRR (95% CI)	All 2 IRR (95% CI)
Early neon. deaths	Never-married	1.44 (1.36, 1.52)	1.44 (1.36, 1.52)	1.44 (1.36, 1.52)	1.44 (1.36, 1.52)
	Widowed	2.53 (1.77, 3.63)	2.36 (1.63, 3.43)	2.66 (1.87, 3.78)	2.95 (2.12, 4.12)
	Divorced	1.89 (1.69, 2.11)	1.89 (1.69, 2.11)	1.89 (1.69, 2.11)	1.89 (1.69, 2.11)
Perinatal deaths	Never-married	1.82 (1.77, 1.88)	1.82 (1.77, 1.88)	1.82 (1.77, 1.88)	1.82 (1.77, 1.88)
	Widowed	1.86 (1.45, 2.40)	1.68 (1.29, 2.19)	1.85 (1.43, 2.38)	2.02 (1.58, 2.57)
	Divorced	1.21 (1.11, 1.31)	1.21 (1.11, 1.31)	1.21 (1.11, 1.31)	1.21 (1.11, 1.31)

Notes: Mortality differences by maternal marital status were estimated using quasi-Poisson regression with marital status as a categorical predictor. Calendar year was not included in the regression models. In the binary analysis (Panel A), married mothers constituted the reference category and never-married, widowed, and divorced mothers were combined into a 'not married' category. In the disaggregated analysis (Panel B), married mothers constituted the reference category for comparisons with never-married, widowed, and divorced mothers. The logarithm of total births (live births plus late fetal deaths) was used as the offset for late fetal and perinatal mortality. In contrast, the logarithm of live births was used for early neonatal mortality. Incidence rate ratios (IRRs) are presented with 95% confidence intervals (CIs). To assess the potential influence of small-cell reconstruction, a conservative sensitivity analysis was performed at the level of the original four marital-status categories. All

completed cells with death counts of 1 or 2 were treated as potentially affected by source-data suppression and were simultaneously assigned values of 1, 1.5, or 2. For the binary analysis, the four categories were aggregated into married and not married only after applying these alternative values. Cells with a value of 0 and cells with counts ≥3 were left unchanged. The ‘Current’ column represents estimates obtained from the dataset used in the primary analysis. The alternative scenarios are intended as sensitivity bounds and do not imply that all cells with completed values of 1 or 2 were originally suppressed or required estimation.

Table S12. Annual trends in late fetal, early neonatal, and perinatal mortality in Hungary, 2000–2023.

Maternal age	Late fetal mortality<br>IRR (95% CI)	Early neon. mortality<br>IRR (95% CI)	Perinatal mortality<br>IRR (95% CI)
Adolescents (under 20 years)	1.01 (1.00, 1.02)	0.96 (0.95, 0.97)	0.99 (0.98, 1.00)
Optimal reproductive years (20–34 years)	0.99 (0.99, 1.00)	0.95 (0.94, 0.95)	0.98 (0.98, 0.98)
Advanced maternal age (35–49 years)	0.96 (0.96, 0.97)	0.92 (0.91, 0.93)	0.95 (0.94, 0.95)

Notes: Incidence rate ratios (IRRs) were estimated using quasi-Poisson regression models with live total births (late fetal mortality, perinatal mortality) and live births (early neonatal mortality) as offset terms, respectively.

Table S13. Differences due to maternal age in late fetal, early neonatal, and perinatal mortality in Hungary, 2000–2023 (reference: 20–34 years).

Maternal age	Late fetal mortality IRR (95% CI)	Early neon. mortality IRR (95% CI)	Perinatal mortality IRR (95% CI)
Adolescents (under 20 years)	1.55 (1.45, 1.65)	1.41 (1.27, 1.55)	1.50 (1.42, 1.58)
Optimal reproductive years (20–34 years)	1.00 (Reference)	1.00 (Reference)	1.00 (Reference)
Advanced maternal age (35–49 years)	1.27 (1.21, 1.33)	1.22 (1.14, 1.31)	1.26 (1.21, 1.31)

Notes: Incidence rate ratios (IRRs) were estimated using quasi-Poisson regression models with live total births (late fetal mortality, perinatal mortality) and live births (early neonatal mortality) as offset terms, respectively.

Table S14. Differences due to maternal age in late fetal, early neonatal, and perinatal mortality in Hungary, 2000–2023 (reference: 25–29 years).

Maternal age	Late fetal mortality IRR (95% CI)	Early neon. mortality IRR (95% CI)	Perinatal mortality IRR (95% CI)
Under 15 years	1.10 (0.57, 2.12)	1.42 (0.64, 3.18)	1.21 (0.73, 2.01)
15–19 years	1.66 (1.54, 1.79)	1.49 (1.34, 1.66)	1.60 (1.51, 1.70)
20–24 years	1.35 (1.28, 1.43)	1.17 (1.08, 1.27)	1.29 (1.23, 1.35)
25–29 years	1.00 (Reference)	1.00 (Reference)	1.00 (Reference)
30–34 years	0.98 (0.93, 1.04)	1.05 (0.98, 1.13)	1.00 (0.97, 1.05)
35–39 years	1.24 (1.17, 1.32)	1.23 (1.13, 1.34)	1.23 (1.17, 1.30)
40–44 years	1.90 (1.73, 2.08)	1.59 (1.38, 1.83)	1.79 (1.66, 1.93)
45–49 years	2.20 (1.52, 3.17)	1.77 (1.00, 3.12)	2.05 (1.51, 2.78)

Notes: Incidence rate ratios (IRRs) were estimated using quasi-Poisson regression models with live total births (late fetal mortality, perinatal mortality) and live births (early neonatal mortality) as offset terms, respectively.

Table S15. Sensitivity analysis of mortality differences across broad maternal-age categories under alternative assumptions for potentially suppressed small cell counts, Hungary, 2000–2023.

Mortality group	Maternal age	Current IRR (95% CI)	All 1 IRR (95% CI)	All 1.5 IRR (95% CI)	All 2 IRR (95% CI)
Late fetal deaths	0–19	1.55 (1.45, 1.65)	1.55 (1.45, 1.65)	1.55 (1.45, 1.66)	1.56 (1.46, 1.67)
	35–49	1.27 (1.21, 1.33)	1.27 (1.21, 1.33)	1.27 (1.21, 1.33)	1.28 (1.22, 1.34)
Early neon. deaths	0–19	1.41 (1.27, 1.55)	1.41 (1.27, 1.55)	1.41 (1.28, 1.56)	1.42 (1.28, 1.56)
	35–49	1.22 (1.14, 1.31)	1.22 (1.14, 1.31)	1.23 (1.15, 1.31)	1.23 (1.15, 1.32)
Perinatal deaths	0–19	1.50 (1.42, 1.58)	1.50 (1.42, 1.58)	1.50 (1.42, 1.59)	1.51 (1.43, 1.60)
	35–49	1.26 (1.21, 1.31)	1.25 (1.20, 1.30)	1.26 (1.21, 1.31)	1.26 (1.21, 1.31)

Notes: Mortality differences across maternal-age categories were estimated using quasi-Poisson regression with maternal age as a categorical predictor and 20–34 years as the reference category. Calendar year was not included in the regression models. The logarithm of total births (live births plus late fetal deaths) was used as the offset for late fetal and perinatal mortality. In contrast, the logarithm of live births was used for early neonatal mortality. Incidence rate ratios (IRRs) are presented with 95% confidence intervals (CIs). To assess the potential influence of small-cell reconstruction, a conservative sensitivity analysis was performed at the level of the original eight maternal-age categories. All completed cells with death counts of 1 or 2 were treated as potentially affected by source-data

suppression and were simultaneously assigned values of 1, 1.5, or 2 before aggregation into the three broad maternal-age categories (<20, 20–34, and ≥35 years). Cells with a value of 0 and cells with counts ≥3 were left unchanged. The 'Current' column represents estimates obtained from the dataset used in the primary analysis. The alternative scenarios are intended as sensitivity bounds and do not imply that all cells with completed values of 1 or 2 were originally suppressed or required estimation.

Table S16. Sensitivity analysis of mortality differences across detailed maternal-age categories under alternative assumptions for potentially suppressed small cell counts, Hungary, 2000–2023.

Mortality group	Maternal age	Current IRR (95% CI)	All 1 IRR (95% CI)	All 1.5 IRR (95% CI)	All 2 IRR (95% CI)
Late fetal deaths	0–14	1.10 (0.57, 2.12)	1.10 (0.57, 2.12)	1.65 (0.97, 2.82)	2.21 (1.39, 3.51)
	15–19	1.66 (1.54, 1.79)	1.66 (1.54, 1.79)	1.66 (1.54, 1.79)	1.66 (1.54, 1.79)
	20–24	1.35 (1.28, 1.43)	1.35 (1.28, 1.43)	1.35 (1.28, 1.43)	1.35 (1.28, 1.43)
	30–34	0.98 (0.93, 1.04)	0.98 (0.93, 1.04)	0.98 (0.93, 1.04)	0.98 (0.93, 1.04)
	35–39	1.24 (1.17, 1.32)	1.24 (1.17, 1.32)	1.24 (1.17, 1.32)	1.24 (1.17, 1.32)
	40–44	1.90 (1.73, 2.08)	1.90 (1.73, 2.08)	1.90 (1.73, 2.08)	1.90 (1.73, 2.08)
	45–49	2.20 (1.52, 3.17)	1.82 (1.22, 2.72)	2.27 (1.59, 3.26)	2.73 (1.96, 3.79)
Early neon. deaths	0–14	1.42 (0.64, 3.18)	1.42 (0.64, 3.18)	1.78 (0.87, 3.65)	2.14 (1.11, 4.12)
	15–19	1.49 (1.34, 1.66)	1.49 (1.34, 1.66)	1.49 (1.34, 1.66)	1.49 (1.34, 1.66)
	20–24	1.17 (1.08, 1.27)	1.17 (1.08, 1.27)	1.17 (1.08, 1.27)	1.17 (1.08, 1.27)

Mortality group	Maternal age	Current IRR (95% CI)	All 1 IRR (95% CI)	All 1.5 IRR (95% CI)	All 2 IRR (95% CI)
	30–34	1.05 (0.98, 1.13)	1.05 (0.98, 1.13)	1.05 (0.98, 1.13)	1.05 (0.98, 1.13)
	35–39	1.23 (1.13, 1.34)	1.23 (1.13, 1.34)	1.23 (1.13, 1.34)	1.23 (1.13, 1.34)
	40–44	1.59 (1.38, 1.83)	1.59 (1.38, 1.83)	1.59 (1.38, 1.83)	1.59 (1.38, 1.83)
	45–49	1.77 (1.00, 3.12)	1.62 (0.90, 2.93)	2.21 (1.33, 3.68)	2.80 (1.78, 4.40)
Perinatal deaths	0–14	1.21 (0.73, 2.01)	1.21 (0.73, 2.01)	1.70 (1.11, 2.61)	2.18 (1.49, 3.19)
	15–19	1.60 (1.51, 1.70)	1.60 (1.51, 1.70)	1.60 (1.51, 1.70)	1.60 (1.51, 1.70)
	20–24	1.29 (1.23, 1.35)	1.29 (1.23, 1.35)	1.29 (1.23, 1.35)	1.29 (1.23, 1.35)
	30–34	1.01 (0.97, 1.05)	1.01 (0.97, 1.05)	1.01 (0.97, 1.05)	1.01 (0.97, 1.05)
	35–39	1.23 (1.17, 1.30)	1.23 (1.17, 1.30)	1.23 (1.17, 1.30)	1.23 (1.17, 1.30)
	40–44	1.79 (1.66, 1.93)	1.79 (1.66, 1.93)	1.79 (1.66, 1.93)	1.79 (1.66, 1.93)
	45–49	2.05 (1.51, 2.78)	1.65 (1.17, 2.32)	2.02 (1.48, 2.76)	2.40 (1.80, 3.19)

Notes: Mortality differences across the eight maternal-age categories (0–14, 15–19, 20–24, 25–29, 30–34, 35–39, 40–44, and 45–49 years) were estimated using quasi-Poisson regression with maternal age as a categorical predictor and 25–29 years as the reference category. Calendar year was not included in the regression models. The logarithm of total births (live births plus late fetal deaths) was used as the offset for late fetal and perinatal mortality. In contrast, the logarithm of live births was used for early neonatal mortality. Incidence rate ratios (IRRs) are presented with 95% confidence intervals (CIs). To assess the potential influence of small-cell reconstruction, a conservative sensitivity analysis was performed in which all completed cells with death counts of 1 or 2 were treated as potentially affected by source-data suppression and were simultaneously assigned values of 1, 1.5, or 2. Cells with a value of 0 and cells with counts ≥3 were left unchanged. The 'Current' column represents estimates obtained from the dataset used in the primary analysis. The alternative scenarios are intended as sensitivity bounds and do not imply that all cells with completed values of 1 or 2 were originally suppressed or required estimation.

Table S17. Absolute cause-specific mortality rates and proportional contributions of major causes to late fetal, early neonatal, and perinatal mortality by study period, Hungary, 2000–2023.

Mortality group	Cause	2000–2019, n (%)	Rate per 1,000	2020–2023, n (%)	Rate per 1,000
Late fetal deaths	P05	191 (2.1%)	0.10	110 (6.7%)	0.30
	P07	464 (5.1%)	0.25	0 (0%)	0.00
	P20–P21	3,056 (33.8%)	1.62	698 (42.3%)	1.93
	Q00–Q99	254 (2.8%)	0.13	68 (4.1%)	0.19
Early neonatal deaths	P05	19 (0.4%)	0.01	3 (0.6%)	0.01
	P07	1,032 (21.4%)	0.55	95 (18.6%)	0.26
	P20–P21	305 (6.3%)	0.16	57 (11.2%)	0.16
	Q00–Q99	940 (19.4%)	0.50	109 (21.4%)	0.30
Perinatal deaths	P05	210 (1.5%)	0.11	113 (5.2%)	0.31
	P07	1,496 (10.8%)	0.79	95 (4.4%)	0.26
	P20–P21	3,361 (24.2%)	1.78	755 (35%)	2.09
	Q00–Q99	1,194 (8.6%)	0.63	177 (8.2%)	0.49

Notes: Data are presented as the number of cause-specific deaths (n), the proportion of deaths attributed to each cause within the respective mortality outcome (%), and the cause-specific mortality rate per 1,000 births. For late fetal and perinatal mortality, rates were calculated using total births as the denominator; for early neonatal mortality, rates were calculated using live births as the denominator. Because the two study periods differ in duration, numbers of deaths should not be directly compared between periods. P05, fetal growth restriction and malnutrition; P07, disorders related to short gestation and low birth weight; P20–P21, intrauterine hypoxia and birth asphyxia; Q00–Q99, congenital anomalies.

Table S18. Absolute cause-specific mortality rates and proportional contributions of other respiratory disorders and unspecified causes to late fetal, early neonatal, and perinatal mortality by study period, Hungary, 2000–2023.

Mortality group	Cause	2000–2019, n (%)	Rate per 1,000	2020–2023, n (%)	Rate per 1,000
Late fetal deaths	P24–P28	160 (1.8%)	0.08	1 (0.1%)	0.00
	Unspec.	4,812 (53.2%)	2.55	750 (45.5%)	2.08
Early neonatal deaths	P24–P28	610 (12.6%)	0.33	26 (5.1%)	0.07
	Unspec.	141 (2.9%)	0.08	52 (10.2%)	0.14
Perinatal deaths	P24–P28	770 (5.5%)	0.41	27 (1.3%)	0.07
	Unspec.	4,953 (35.7%)	2.63	802 (37.1%)	2.22

Notes: Data are presented as the number of cause-specific deaths (n), the proportion of deaths attributed to each cause within the respective mortality outcome (%), and the cause-specific mortality rate per 1,000 births. For late fetal and perinatal mortality, rates were calculated using total births as the denominator; for early neonatal mortality, rates were calculated using live births as the denominator. Because the two study periods differ in duration, numbers of deaths should not be directly compared between periods. P24–P28, other respiratory conditions originating in the perinatal period; Unspecified, other unspecified conditions.

Table S19. Sensitivity analysis of cause-specific annual mortality trends under alternative assumptions for potentially suppressed small cell counts, Hungary, 2000–2023.

Mortality group	Death cause	Current IRR (95% CI)	All 1 IRR (95% CI)	All 1.5 IRR (95% CI)	All 2 IRR (95% CI)
Late fetal deaths	P05	1.14 (1.12, 1.16)	1.14 (1.12, 1.17)	1.14 (1.12, 1.16)	1.13 (1.11, 1.16)
	P07	0.89 (0.88, 0.91)	0.89 (0.88, 0.91)	0.89 (0.88, 0.91)	0.89 (0.88, 0.91)
	P20–P21	1.02 (1.02, 1.03)	1.02 (1.02, 1.03)	1.02 (1.02, 1.03)	1.02 (1.02, 1.03)
	Q00–Q99	1.06 (1.05, 1.08)	1.06 (1.05, 1.08)	1.06 (1.05, 1.08)	1.06 (1.04, 1.08)
Early neon. deaths	P05	0.87 (0.81, 0.94)	0.90 (0.83, 0.97)	0.92 (0.87, 0.98)	0.94 (0.89, 0.99)
	P07	0.88 (0.87, 0.89)	0.88 (0.87, 0.89)	0.88 (0.87, 0.89)	0.88 (0.87, 0.89)
	P20–P21	0.99 (0.97, 1.00)	0.99 (0.97, 1.00)	0.99 (0.97, 1.00)	0.99 (0.97, 1.00)
	Q00–Q99	0.97 (0.96, 0.97)	0.97 (0.96, 0.97)	0.97 (0.96, 0.97)	0.97 (0.96, 0.97)
Perinatal deaths	P05	1.12 (1.10, 1.14)	1.12 (1.10, 1.14)	1.12 (1.10, 1.14)	1.11 (1.09, 1.13)
	P07	0.88 (0.87, 0.89)	0.88 (0.87, 0.89)	0.88 (0.87, 0.89)	0.88 (0.87, 0.89)

Mortality group	Death cause	Current IRR (95% CI)	All 1 IRR (95% CI)	All 1.5 IRR (95% CI)	All 2 IRR (95% CI)
	P20–P21	1.02 (1.01, 1.02)	1.02 (1.01, 1.02)	1.02 (1.01, 1.02)	1.02 (1.01, 1.02)
	Q00–Q99	0.99 (0.98, 1.00)	0.99 (0.98, 1.00)	0.99 (0.98, 1.00)	0.99 (0.98, 1.00)

Notes: Annual cause-specific mortality trends were estimated using quasi-Poisson regression with calendar year as a continuous predictor and the logarithm of the corresponding population at risk as an offset. Incidence rate ratios (IRRs) represent the multiplicative change in the cause-specific mortality rate per calendar year and are presented with 95% confidence intervals (CIs). To assess the potential influence of small-cell reconstruction, a conservative sensitivity analysis was performed in which all sex- and year-specific cells with completed counts of 1 or 2 were treated as potentially affected by source-data suppression and were simultaneously assigned values of 1, 1.5, or 2. Cells with a value of 0 and cells with counts ≥3 were left unchanged. The 'Current' column represents estimates obtained from the dataset used in the primary analysis. The alternative scenarios are intended as sensitivity bounds and do not imply that all cells with completed values of 1 or 2 were originally suppressed or required estimation.

Table S20. Sensitivity analysis of regional differences in cause-specific late fetal mortality under alternative assumptions for potentially suppressed small cell counts, Hungary, 2000–2023.

Death cause	NUTS2 region	Current IRR (95% CI)	All 1 IRR (95% CI)	All 1.5 IRR (95% CI)	All 2 IRR (95% CI)
P05	Pest County	0.99 (0.62, 1.58)	1.00 (0.62, 1.63)	1.04 (0.66, 1.65)	1.07 (0.69, 1.66)
	Central Transdanubia	1.83 (1.20, 2.80)	1.85 (1.19, 2.87)	1.81 (1.19, 2.76)	1.77 (1.18, 2.67)
	Western Transdanubia	1.34 (0.83, 2.16)	1.20 (0.72, 2.00)	1.32 (0.82, 2.13)	1.43 (0.91, 2.23)
	Southern Transdanubia	1.02 (0.60, 1.72)	0.94 (0.54, 1.65)	1.00 (0.59, 1.70)	1.06 (0.64, 1.73)
	Northern Hungary	1.74 (1.15, 2.62)	1.81 (1.19, 2.76)	1.77 (1.18, 2.65)	1.73 (1.17, 2.56)
	Northern Great Plain	0.82 (0.52, 1.31)	0.78 (0.48, 1.27)	0.82 (0.52, 1.31)	0.86 (0.56, 1.34)
	Southern Great Plain	1.69 (1.11, 2.57)	1.79 (1.17, 2.75)	1.69 (1.11, 2.56)	1.60 (1.07, 2.39)
P07	Pest County	1.16 (0.78, 1.71)	1.13 (0.77, 1.68)	1.16 (0.79, 1.71)	1.18 (0.81, 1.74)
	Central Transdanubia	1.47 (1.00, 2.17)	1.44 (0.98, 2.13)	1.49 (1.01, 2.19)	1.53 (1.05, 2.25)
	Western Transdanubia	1.29 (0.85, 1.96)	1.18 (0.77, 1.82)	1.26 (0.83, 1.92)	1.33 (0.88, 2.01)

Death cause	NUTS2 region	Current IRR (95% CI)	All 1 IRR (95% CI)	All 1.5 IRR (95% CI)	All 2 IRR (95% CI)
	Southern Transdanubia	0.99 (0.62, 1.57)	0.95 (0.59, 1.52)	1.05 (0.67, 1.65)	1.15 (0.74, 1.77)
	Northern Hungary	3.32 (2.42, 4.57)	3.27 (2.38, 4.50)	3.27 (2.38, 4.49)	3.26 (2.38, 4.47)
	Northern Great Plain	0.56 (0.36, 0.88)	0.45 (0.27, 0.73)	0.53 (0.33, 0.84)	0.61 (0.39, 0.94)
	Southern Great Plain	2.19 (1.55, 3.09)	2.16 (1.53, 3.05)	2.15 (1.53, 3.04)	2.15 (1.53, 3.02)
P20-P21	Pest County	1.60 (1.33, 1.92)	1.61 (1.34, 1.94)	1.60 (1.33, 1.92)	1.58 (1.31, 1.90)
	Central Transdanubia	3.50 (2.96, 4.13)	3.53 (2.99, 4.18)	3.49 (2.95, 4.12)	3.45 (2.92, 4.07)
	Western Transdanubia	2.07 (1.71, 2.51)	2.10 (1.73, 2.54)	2.07 (1.71, 2.50)	2.04 (1.69, 2.47)
	Southern Transdanubia	2.22 (1.84, 2.69)	2.25 (1.86, 2.72)	2.22 (1.84, 2.68)	2.19 (1.81, 2.65)
	Northern Hungary	7.58 (6.52, 8.81)	7.66 (6.59, 8.90)	7.56 (6.51, 8.78)	7.47 (6.43, 8.67)
	Northern Great Plain	4.56 (3.91, 5.32)	4.61 (3.95, 5.38)	4.55 (3.90, 5.30)	4.50 (3.86, 5.24)
	Southern Great Plain	2.40 (2.02, 2.86)	2.43 (2.04, 2.89)	2.40 (2.01, 2.85)	2.37 (1.99, 2.81)

Death cause	NUTS2 region	Current IRR (95% CI)	All 1 IRR (95% CI)	All 1.5 IRR (95% CI)	All 2 IRR (95% CI)
Q00-Q99	Pest County	1.07 (0.71, 1.62)	1.06 (0.69, 1.62)	1.08 (0.72, 1.64)	1.11 (0.75, 1.65)
	Central Transdanubia	1.34 (0.89, 2.02)	1.28 (0.83, 1.98)	1.32 (0.87, 2.00)	1.35 (0.91, 2.01)
	Western Transdanubia	1.14 (0.73, 1.79)	1.04 (0.64, 1.69)	1.14 (0.72, 1.78)	1.22 (0.79, 1.87)
	Southern Transdanubia	1.01 (0.63, 1.62)	0.84 (0.50, 1.42)	1.00 (0.62, 1.61)	1.13 (0.73, 1.76)
	Northern Hungary	1.08 (0.71, 1.63)	1.03 (0.66, 1.59)	1.06 (0.70, 1.61)	1.09 (0.73, 1.63)
	Northern Great Plain	0.64 (0.41, 1.00)	0.65 (0.41, 1.03)	0.67 (0.43, 1.05)	0.70 (0.46, 1.06)
	Southern Great Plain	1.60 (1.10, 2.34)	1.65 (1.12, 2.44)	1.63 (1.12, 2.38)	1.61 (1.12, 2.33)

Notes: Regional differences in cause-specific late fetal mortality were estimated using quasi-Poisson regression with region as a categorical predictor, Budapest as the reference category, and the logarithm of total births (live births plus late fetal deaths) as an offset. Incidence rate ratios (IRRs) are presented with 95% confidence intervals (CIs). To assess the potential influence of small-cell reconstruction, a conservative sensitivity analysis was performed in which all completed cells with counts of 1 or 2 were treated as potentially affected by source-data suppression and were simultaneously assigned values of 1, 1.5, or 2. Cells with a value of 0 and cells with counts ≥3 were left unchanged. The 'Current' column represents estimates obtained from the dataset used in the primary

analysis. The alternative scenarios are intended as sensitivity bounds and do not imply that all cells with completed values of 1 or 2 were originally suppressed or required estimation.

Table S21. Sensitivity analysis of regional differences in cause-specific early neonatal mortality under alternative assumptions for potentially suppressed small cell counts, Hungary, 2000–2023.

Death cause	NUTS2 region	Current IRR (95% CI)	All 1 IRR (95% CI)	All 1.5 IRR (95% CI)	All 2 IRR (95% CI)
P05	Pest County	0.85 (0.14, 5.10)	0.85 (0.14, 5.10)	1.28 (0.26, 6.33)	1.70 (0.38, 7.62)
	Central Transdanubia	1.06 (0.18, 6.37)	0.53 (0.06, 5.11)	0.80 (0.11, 5.66)	1.06 (0.18, 6.37)
	Western Transdanubia	1.23 (0.21, 7.36)	1.23 (0.21, 7.36)	1.85 (0.37, 9.15)	2.46 (0.55, 11.00)
	Southern Transdanubia	0.65 (0.07, 6.21)	0.65 (0.07, 6.21)	0.97 (0.14, 6.88)	1.29 (0.22, 7.73)
	Northern Hungary	1.32 (0.27, 6.53)	0.88 (0.15, 5.26)	1.32 (0.27, 6.53)	1.76 (0.39, 7.85)
	Northern Great Plain	2.06 (0.51, 8.22)	2.06 (0.51, 8.22)	2.40 (0.62, 9.28)	2.74 (0.73, 10.34)
	Southern Great Plain	2.30 (0.55, 9.64)	2.30 (0.55, 9.64)	3.46 (0.91, 13.18)	4.61 (1.27, 16.74)
P07	Pest County	1.26 (1.01, 1.58)	1.29 (1.03, 1.61)	1.29 (1.03, 1.61)	1.29 (1.04, 1.61)
	Central Transdanubia	1.14 (0.89, 1.45)	1.14 (0.89, 1.46)	1.18 (0.93, 1.50)	1.21 (0.96, 1.54)
	Western Transdanubia	1.74 (1.39, 2.18)	1.72 (1.37, 2.17)	1.74 (1.39, 2.18)	1.75 (1.40, 2.19)

Death cause	NUTS2 region	Current IRR (95% CI)	All 1 IRR (95% CI)	All 1.5 IRR (95% CI)	All 2 IRR (95% CI)
	Southern Transdanubia	1.62 (1.28, 2.04)	1.60 (1.27, 2.03)	1.62 (1.28, 2.04)	1.63 (1.30, 2.05)
	Northern Hungary	1.07 (0.84, 1.35)	1.06 (0.83, 1.34)	1.06 (0.84, 1.35)	1.07 (0.85, 1.35)
	Northern Great Plain	0.86 (0.68, 1.08)	0.88 (0.69, 1.11)	0.89 (0.70, 1.12)	0.89 (0.71, 1.12)
	Southern Great Plain	1.38 (1.11, 1.72)	1.39 (1.11, 1.74)	1.40 (1.12, 1.75)	1.41 (1.13, 1.75)
P20-P21	Pest County	1.11 (0.73, 1.70)	0.95 (0.60, 1.50)	1.04 (0.68, 1.58)	1.11 (0.75, 1.65)
	Central Transdanubia	1.01 (0.63, 1.60)	0.85 (0.51, 1.42)	1.05 (0.67, 1.64)	1.20 (0.80, 1.82)
	Western Transdanubia	1.20 (0.76, 1.91)	0.99 (0.60, 1.64)	1.10 (0.69, 1.74)	1.18 (0.77, 1.82)
	Southern Transdanubia	1.22 (0.77, 1.94)	1.04 (0.62, 1.72)	1.17 (0.74, 1.86)	1.28 (0.83, 1.96)
	Northern Hungary	2.15 (1.49, 3.10)	2.21 (1.51, 3.22)	2.07 (1.44, 2.98)	1.96 (1.39, 2.78)
	Northern Great Plain	1.43 (0.98, 2.09)	1.39 (0.93, 2.06)	1.37 (0.94, 1.99)	1.36 (0.95, 1.94)
	Southern Great Plain	1.26 (0.83, 1.92)	1.19 (0.77, 1.85)	1.22 (0.81, 1.85)	1.25 (0.85, 1.85)

Death cause	NUTS2 region	Current IRR (95% CI)	All 1 IRR (95% CI)	All 1.5 IRR (95% CI)	All 2 IRR (95% CI)
Q00-Q99	Pest County	0.89 (0.70, 1.13)	0.85 (0.66, 1.09)	0.86 (0.68, 1.11)	0.88 (0.69, 1.12)
	Central Transdanubia	1.06 (0.82, 1.35)	1.04 (0.81, 1.34)	1.06 (0.83, 1.36)	1.07 (0.84, 1.37)
	Western Transdanubia	1.00 (0.77, 1.30)	0.97 (0.74, 1.27)	1.00 (0.77, 1.30)	1.03 (0.80, 1.34)
	Southern Transdanubia	1.39 (1.09, 1.78)	1.34 (1.05, 1.71)	1.36 (1.07, 1.73)	1.38 (1.08, 1.75)
	Northern Hungary	1.53 (1.23, 1.89)	1.54 (1.24, 1.90)	1.52 (1.23, 1.89)	1.51 (1.22, 1.87)
	Northern Great Plain	1.00 (0.80, 1.24)	1.00 (0.80, 1.25)	1.00 (0.80, 1.24)	0.99 (0.79, 1.24)
	Southern Great Plain	1.25 (1.00, 1.57)	1.23 (0.98, 1.55)	1.23 (0.98, 1.55)	1.23 (0.98, 1.55)

Notes: Regional differences in cause-specific early neonatal mortality were estimated using quasi-Poisson regression with region as a categorical predictor, Budapest as the reference category, and the logarithm of live births as an offset. Incidence rate ratios (IRRs) are presented with 95% confidence intervals (CIs). To assess the potential influence of small-cell reconstruction, a conservative sensitivity analysis was performed in which all completed cells with counts of 1 or 2 were treated as potentially affected by source-data suppression and were simultaneously assigned values of 1, 1.5, or 2. Cells with a value of 0 and cells with counts ≥3 were left unchanged. The 'Current' column represents estimates obtained from the dataset used in the primary analysis. The alternative scenarios are intended as

sensitivity bounds and do not imply that all cells with completed values of 1 or 2 were originally suppressed or required estimation.

Table S22. Sensitivity analysis of regional differences in cause-specific perinatal mortality under alternative assumptions for potentially suppressed small cell counts, Hungary, 2000–2023.

Death cause	NUTS2 region	Current IRR (95% CI)	All 1 IRR (95% CI)	All 1.5 IRR (95% CI)	All 2 IRR (95% CI)
P05	Pest County	0.98 (0.62, 1.54)	0.96 (0.60, 1.54)	1.01 (0.65, 1.58)	1.06 (0.69, 1.62)
	Central Transdanubia	1.78 (1.18, 2.68)	1.75 (1.14, 2.69)	1.74 (1.15, 2.62)	1.73 (1.16, 2.57)
	Western Transdanubia	1.33 (0.84, 2.11)	1.24 (0.76, 2.03)	1.36 (0.86, 2.14)	1.45 (0.94, 2.23)
	Southern Transdanubia	0.99 (0.59, 1.65)	0.92 (0.53, 1.59)	1.00 (0.60, 1.66)	1.07 (0.66, 1.73)
	Northern Hungary	1.71 (1.15, 2.55)	1.77 (1.18, 2.67)	1.74 (1.17, 2.57)	1.71 (1.17, 2.49)
	Northern Great Plain	0.91 (0.59, 1.40)	0.87 (0.55, 1.38)	0.93 (0.61, 1.43)	0.98 (0.65, 1.48)
	Southern Great Plain	1.73 (1.16, 2.59)	1.79 (1.19, 2.71)	1.71 (1.15, 2.55)	1.64 (1.12, 2.42)
P07	Pest County	1.24 (1.02, 1.50)	1.26 (1.04, 1.53)	1.26 (1.04, 1.52)	1.25 (1.04, 1.52)
	Central Transdanubia	1.22 (0.99, 1.50)	1.25 (1.02, 1.54)	1.26 (1.02, 1.54)	1.26 (1.03, 1.55)
	Western Transdanubia	1.62 (1.33, 1.98)	1.62 (1.32, 1.98)	1.62 (1.33, 1.97)	1.62 (1.33, 1.97)

**Death cause**	**NUTS2 region**	**Current IRR (95% CI)**	**All 1 IRR (95% CI)**	**All 1.5 IRR (95% CI)**	**All 2 IRR (95% CI)**
	Southern Transdanubia	1.46 (1.18, 1.79)	1.48 (1.20, 1.82)	1.48 (1.20, 1.82)	1.48 (1.20, 1.81)
	Northern Hungary	1.63 (1.36, 1.96)	1.66 (1.38, 1.99)	1.64 (1.36, 1.96)	1.62 (1.35, 1.94)
	Northern Great Plain	0.78 (0.64, 0.96)	0.80 (0.65, 0.98)	0.80 (0.65, 0.98)	0.79 (0.65, 0.97)
	Southern Great Plain	1.58 (1.32, 1.91)	1.61 (1.34, 1.94)	1.60 (1.33, 1.93)	1.60 (1.33, 1.92)
P20-P21	Pest County	1.51 (1.27, 1.79)	1.51 (1.27, 1.79)	1.51 (1.27, 1.79)	1.51 (1.27, 1.79)
	Central Transdanubia	3.03 (2.60, 3.54)	3.03 (2.60, 3.54)	3.03 (2.60, 3.54)	3.03 (2.60, 3.54)
	Western Transdanubia	1.91 (1.60, 2.28)	1.91 (1.60, 2.28)	1.91 (1.60, 2.28)	1.91 (1.60, 2.28)
	Southern Transdanubia	2.04 (1.71, 2.42)	2.04 (1.71, 2.42)	2.04 (1.71, 2.42)	2.04 (1.71, 2.42)
	Northern Hungary	6.56 (5.72, 7.53)	6.56 (5.72, 7.53)	6.56 (5.72, 7.53)	6.56 (5.72, 7.53)
	Northern Great Plain	3.98 (3.46, 4.57)	3.98 (3.46, 4.57)	3.98 (3.46, 4.57)	3.98 (3.46, 4.57)
	Southern Great Plain	2.19 (1.87, 2.57)	2.19 (1.87, 2.57)	2.19 (1.87, 2.57)	2.19 (1.87, 2.57)

Death cause	NUTS2 region	Current IRR (95% CI)	All 1 IRR (95% CI)	All 1.5 IRR (95% CI)	All 2 IRR (95% CI)
Q00-Q99	Pest County	0.93 (0.76, 1.15)	0.92 (0.75, 1.14)	0.93 (0.75, 1.15)	0.93 (0.76, 1.15)
	Central Transdanubia	1.12 (0.91, 1.39)	1.13 (0.91, 1.40)	1.13 (0.91, 1.40)	1.13 (0.92, 1.40)
	Western Transdanubia	1.03 (0.82, 1.30)	1.00 (0.80, 1.26)	1.02 (0.81, 1.28)	1.04 (0.83, 1.31)
	Southern Transdanubia	1.30 (1.05, 1.61)	1.30 (1.04, 1.61)	1.30 (1.05, 1.61)	1.30 (1.05, 1.61)
	Northern Hungary	1.42 (1.17, 1.71)	1.42 (1.18, 1.72)	1.42 (1.17, 1.71)	1.42 (1.17, 1.71)
	Northern Great Plain	0.91 (0.74, 1.11)	0.91 (0.74, 1.11)	0.91 (0.74, 1.11)	0.91 (0.74, 1.11)
	Southern Great Plain	1.33 (1.10, 1.62)	1.34 (1.10, 1.63)	1.34 (1.10, 1.62)	1.33 (1.10, 1.62)

Notes: Regional differences in cause-specific perinatal mortality were estimated using quasi-Poisson regression with region as a categorical predictor, Budapest as the reference category, and the logarithm of total births (live births plus late fetal deaths) as an offset. Incidence rate ratios (IRRs) are presented with 95% confidence intervals (CIs). To assess the potential influence of small-cell reconstruction, a conservative sensitivity analysis was performed in which all completed cells with counts of 1 or 2 were treated as potentially affected by source-data suppression and were simultaneously assigned values of 1, 1.5, or 2. Cells with a value of 0 and cells with counts ≥3 were left unchanged. The ‘Current’ column represents estimates obtained from the dataset used in the primary

analysis. The alternative scenarios are intended as sensitivity bounds and do not imply that all cells with completed values of 1 or 2 were originally suppressed or required estimation.

Figure S1. Linear and nonlinear fitted temporal trends in late fetal, early neonatal, and perinatal mortality in Hungary, 2000–2023.

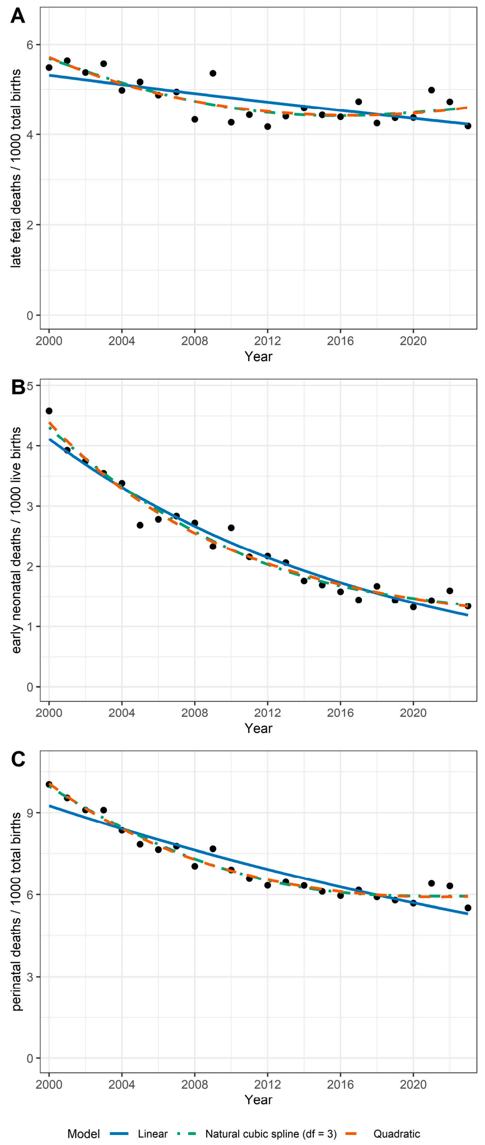

Notes: Observed annual mortality rates are shown as points. Fitted trajectories were obtained from quasi-Poisson models using a linear calendar-year term, a quadratic calendar-year specification, and a natural cubic spline with 3 degrees of freedom, with the logarithm of the relevant population at risk included as an offset. The close agreement between the quadratic and natural cubic spline trajectories indicates that the estimated nonlinear temporal pattern was similar across the two alternative specifications.

Figure S2. Autocorrelation function (ACF) and partial autocorrelation function (PACF) plots of deviance residuals from the quasi-Poisson models of annual late fetal, early neonatal, and perinatal mortality in Hungary, 2000–2023.

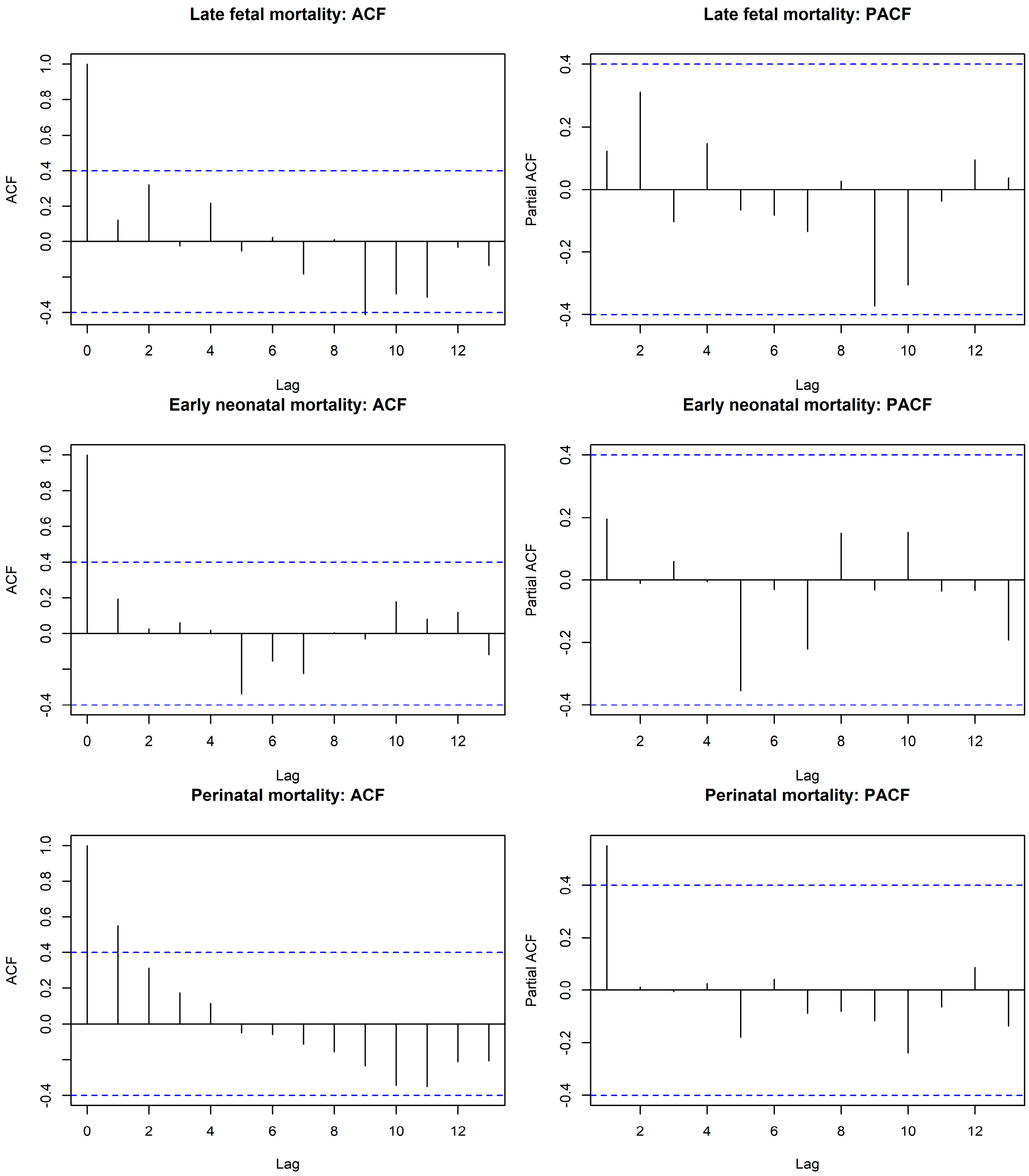

Notes: The plots were used to assess residual temporal autocorrelation in the annual temporal-trend models.

Figure S3. Interrupted time-series analysis of late fetal, early neonatal, and perinatal mortality in Hungary, 2000–2023.

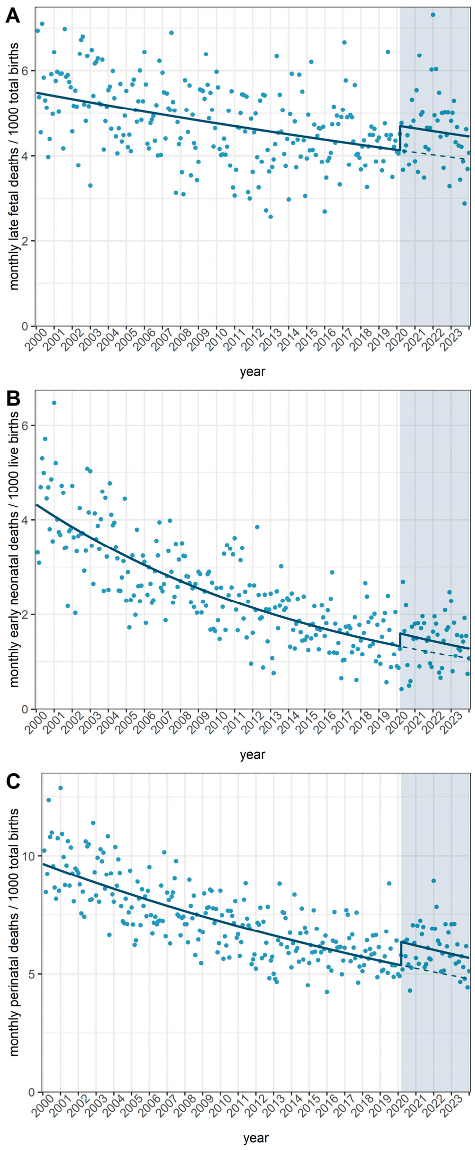

Notes: Monthly observed mortality rates are shown together with fitted model estimates and counterfactual trajectories projected from pre-pandemic trends. March 2020 was specified as the interruption point (shaded area). Counterfactual estimates represent expected mortality in the absence of the COVID-19 pandemic, based on pre-pandemic trends and seasonal patterns derived from quasi-Poisson interrupted time-series models.

Figure S4. Annual trends in perinatal mortality and its components in Hungary, 2000–2023, stratified by sex. (A,B). Late fetal mortality (≥24 gestational weeks), (C,D). early neonatal mortality (0–6 days), (E,F). overall perinatal mortality.

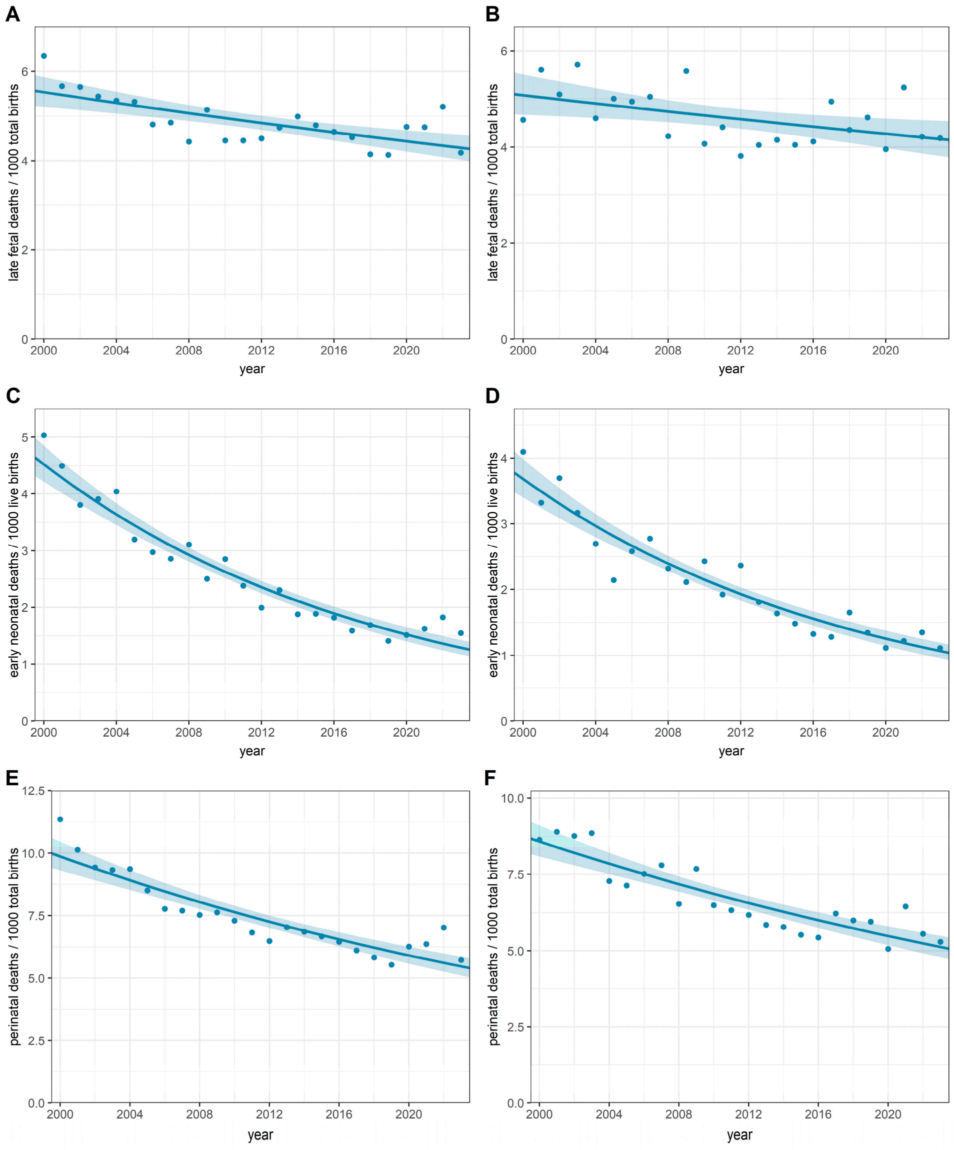

Notes: Lines represent fitted annual trends based on quasi-Poisson regression models with total births (late fetal mortality, perinatal mortality) and live births (early neonatal mortality) as offset terms, respectively, shown separately for males (left panels) and females (right panels).

Figure S5. Regional distribution of perinatal mortality and its components by NUTS 2 regions in Hungary, 2000–2023, stratified by sex. (A,B). Late fetal mortality, (C,D). early neonatal mortality, (E,F). perinatal mortality.

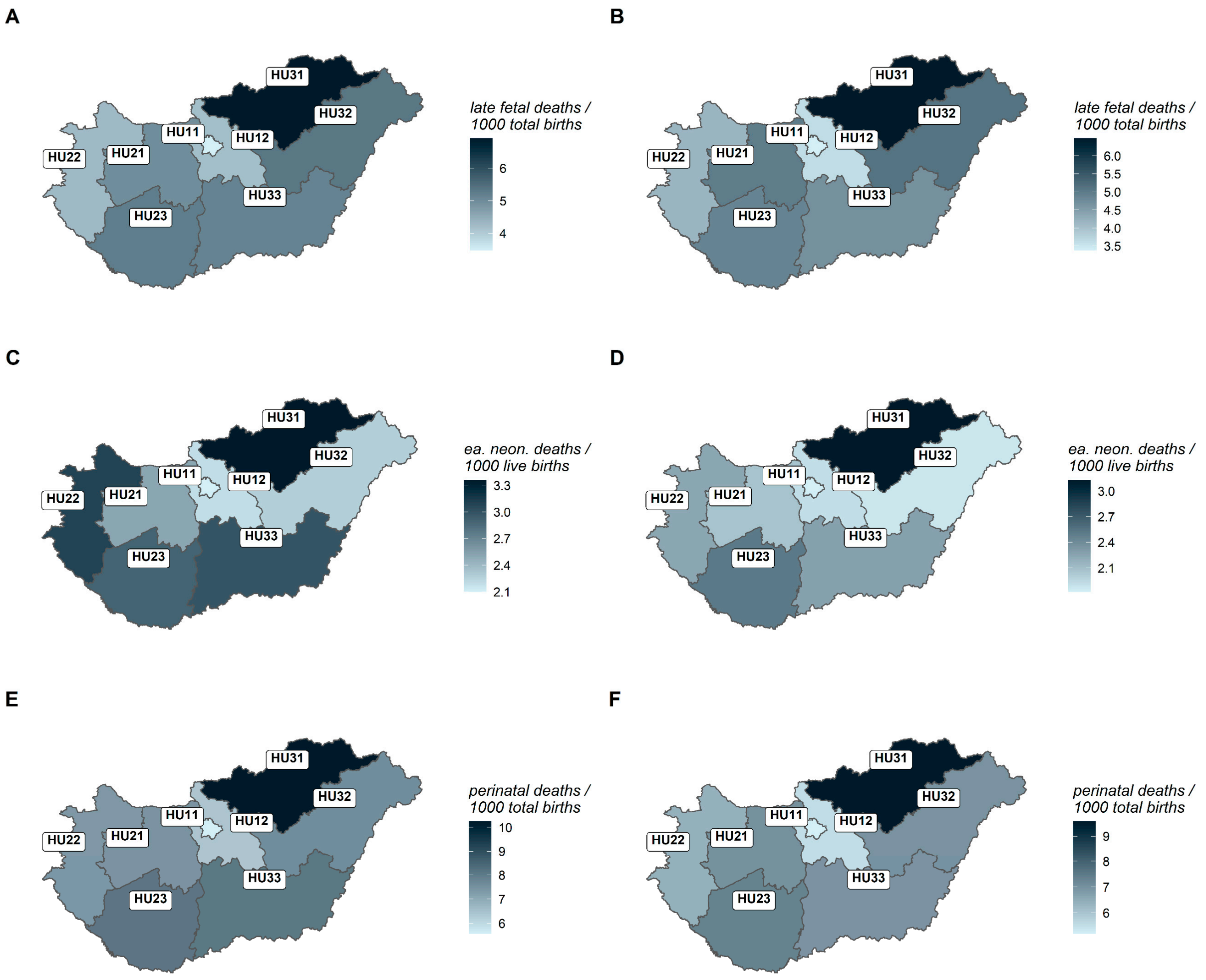

Notes: Mortality rates are presented separately for males (left panels) and females (right panels).
